# Pharmacological mechanisms of Fuzheng Huayu formula for Aristolochic acid I–induced kidney fibrosis through network pharmacology

**DOI:** 10.3389/fphar.2022.1056865

**Published:** 2022-12-08

**Authors:** Fan Wang, Siyuan Wang, Jing Wang, Kai Huang, Gaofeng Chen, Yuan Peng, Chenghai Liu, Yanyan Tao

**Affiliations:** ^1^ Institute of Liver Diseases, Shuguang Hospital affiliated to Shanghai University of Traditional Chinese Medicine, Shanghai, China; ^2^ Shanghai Key Laboratory of Traditional Chinese Clinical Medicine, Shanghai, China; ^3^ Key Laboratory of Liver and Kidney Diseases, Ministry of Education, Shanghai University of Traditional Chinese Medicine, Shanghai, China

**Keywords:** renal fibrosis, Aristolochic acid I, Fuzheng Huayu formula, network pharmacology, mechanisms

## Abstract

Renal fibrosis, characterized by the destruction of renal tubules and interstitial capillaries and the accumulation of extracellular matrix proteins, is a common outcome of chronic renal diseases and has a wide spectrum of etiologies. Fibrosis can affect any organ and has similar pathological mechanisms. Fuzheng Huayu formula (FZHY), as the approved anti-liver fibrosis medicine in China, also can inhibit the kidney fibrosis induced by HgCl_2_ or unilateral ureteral obstruction. However, little is known about the mechanisms underlying the beneficial effects of FZHY on renal fibrosis. This study aimed to identify the mechanisms of FZHY acts on renal fibrosis through network pharmacological analysis and *in vivo* experiments. Data from online databases were mined and screened to predict the target related genes of FZHY acts on renal fibrosis. The STRING and Cytoscape were used to construct the protein-protein interaction (PPI) networks for FZHY and CKD target proteins. Mouse models with CKD induced by Aristolochic Acid I (AAI) were used to validate the effects of FZHY on renal fibrosis and their underlying mechanisms by detecting kidney function, renal fibrosis, and related intersection genes. A total of 129 FZHY–CKD crossover proteins were filtered and constructed into a protein–protein interaction network complex and designated as the potential targets of FZHY. One of the highest-scoring genes, *FOS*, and its related signaling pathways were more activated in CKD. The results demonstrated that FZHY can exert an anti-renal fibrosis effect by improving the levels of serum creatinine and blood urea nitrogen and alleviating excessive collagen deposition in kidney tissue, FZHY also could reduce the levels of *TNF-α*, *IL-1β*, and *IL-6* and inhibit the expression of *MAPK*/*FOS* signal molecules. Our study findings provide insights into predicting the effects of FZHY on CKD through network pharmacology. FZHY can protect the kidney from inflammatory injury caused by AAI and can antagonize inflammatory factor-stimulated *MAPK*/*FOS* activation in fibrotic kidneys. These effects constitute the mechanisms of FZHY for renal fibrosis.

## 1 Introduction

Chronic kidney disease (CKD) is defined as the kidney dysfunction and abnormality. Renal fibrosis is the primary pathological process of CKD and has an overall prevalence of 10.8% in China; however, treatment options are limited (Zhang et al., 2012). Current treatments to reverse the progression of many CKDs are neither effective nor safe enough for clinical application (Klinkhammer et al., 2017). Although angiotensin receptor blockers and angiotensin converting enzyme inhibitors are commonly used together to treat CKD, their effect is unsatisfactory (Qin et al., 2020). One meta-analysis revealed that N-acetylcysteine (NAC) can improve renal function in patients with CKD, and the most frequent side effects are nausea and vomiting (Ye et al., 2021). In a previous study, 11% of stage 3 CKD cases progress into end-stage renal disease (i.e., kidney failure). The only therapeutic regimen for renal disease is dialysis or transplantation (Collins et al., 2015). CKD has also become an urgent public health problem in China. In 2015, 1.06 million patients with incident dialysis were admitted at a cost of 106 billion Chinese Renminbi (Yang et al., 2020)*.*

Traditional Chinese Medicine (TCM) is one therapeutic option for CKD. Several studies have documented that TCM can delay CKD progress and improve patient survival and quality of life (Xi et al., 2020; Xia et al., 2020). The Fuzheng Huayu formula (FZHY) is a well-studied empirical prescription that consists of six Chinese medicinal herbs: *Radix Salvia Miltiorrhizae, Cordyceps, Semen Persicae, Gynostemma Pentaphyllammak, Pollen Pini,* and *Fructus Schisandrae Chinensis*. FZHY was approved by the Chinese State Food and Drug Administration (SFDA) as a drug (No. Z20050547), and it is widely used to treat hepatic fibrosis in China. Additionally, our previous studies have suggested that FZHY significantly decreases kidney collagen deposition and attenuates renal interstitial fibrosis (Wang et al., 2010; Wang et al., 2020). Fibrosis is characterized by dysfunction of capillary networks and the accumulation of fibrillary collagens, which activates myofibroblasts and inflammatory cells (Kang et al., 2015). Similarly, liver fibrosis and kidney fibrosis exhibit common pathological changes. FZHY, an anti-liver fibrotic TCM, also has an anti-renal fibrotic effect. However, the potential therapeutic effect of FZHY on CKD and the pharmacological mechanisms of FZHY require further research.

Network pharmacology can be used to create a complex interactive network based on target molecules, biological functions, and biologically active compounds, which addresses the natural feature of Chinese medicine recipes and systematically reflects the intervention mechanisms of TCM (Luo et al., 2020). In the present study, network pharmacology was used to further elucidate potential target genes and signaling pathways of FZHY for renal fibrosis. Furthermore, the molecular mechanisms underlying the effects of FZHY on renal fibrosis were examined using *in vivo* experiments.

## 2 Materials and methods

### 2.1 Prediction of chronic kidney disease-Related genes from Gene Expression Omnibus profiles

The raw data of renal biopsy from 53 patients with CKD were obtained from the Gene Expression Omnibus under GEO Series (GSE) (accession number: GSE66494) (Nakagaw et al., 2015). The eight normal human adult genome, retrieved from GPL6480, were used as the control.

By incorporating the Affy and Limma software packages in the R language (Ritchie et al., 2015), differentially expressed genes (DEGs) of patients with and without CKD were analyzed. The Affy package removes systematic errors in the original data to obtain standardized gene expression data, and the Limma package is used for the differential expression analysis of normal tissue and kidney injury tissue data after normalization. An adjusted *p*-value of <0.05 and a log_2_|fold change (FC)| ≥ 1.2 were selected as the cutoff criteria. Finally, the upregulated and downregulated differential genes were identified. Finally, the Pheatmap package was used to draw heat maps and volcano maps.

### 2.2 Identification of associated molecular targets of Fuzheng Huayu

The potential molecular targets of FZHY were predicted using the Traditional Chinese Medicine Systems Pharmacology (TCMSP**)** (Ru et al., 2014), BATMAN (Liu et al., 2016), and Drug Bank (Wishart et al., 2018) databases. The target information of each ingredient was retrieved from TCMSP and Drug Bank to construct the potential target group of the active ingredients of FZHY. The meridian information of each medicinal component in the prescription was obtained from the 2015 edition of the *Chinese Pharmacopoeia*. Candidate components were screened and selected based on the following two parameters to evaluate the levels of oral absorption, usability, and biological activity: 1) oral bioavailability of ≥30% and 2) druglikeness of ≥0.18 (Xu et al., 2020).

### 2.3 Screening of targets associated with chronic kidney disease

Information on the various genes associated with CKD was obtained from the GeneCards database (Stelzer et al., 2016) and the Online Mendelian Inheritance in Man (OMIM) database (Amberger & Hamosh, 2017), which are databases for human genes and genetic disorders. The keywords searched in the three databases were “Renal interstitial fibrosis,” “Glomerulosclerosis” and “renal failure” and the results were exported online.

### 2.4 Fuzheng Huayu component-chronic kidney disease target protein–protein interaction network construction

To clarify the interaction between FZHY-related targets and CKD targets, FZHY multicomponent target genes and CKD target genes were genetically deduplicated. The R language was used to program the intersection of the two targets and create the Venn diagram. The intersection targets were submitted to the STRING11.0 database (Szklarczy et al., 2017), where the parameter related to the organism was set to “*Homo sapiens*,” other basic settings were set to default values, and the isolated proteins were hidden. The data were analyzed and exported to the protein–protein interaction (PPI) network diagram. The PPI analysis model was limited to “multiple proteins,” the species was set as “*Homo sapiens*,” and the minimum required interaction score was set to 0.4 with a confidence of ≥0.7 to hide isolated proteins.

### 2.5 Gene ontology functional enrichment analysis and Kyoto Encyclopedia of Genes and Genomes pathway enrichment analysis

To determine the target genes associated with functional annotations and pathway enrichment, gene ontology (GO) annotation and Kyoto Encyclopedia of Genes and Genomes (KEGG) analysis were used. The GO annotation shows the molecular function (MF), biological process (BP), and cellular components (CC) of target genes. Additionally, the pathway enrichment analysis was conducted using the clusterprofiler package (Zhou et al., 2019), and the results were visualized using the enrichplot and DOSE Bioconductor packages (Yu et al., 2015). Enrichment with a *p*-value of <0.05 was considered statistically significant, and the adjusted *p*-value of <0.05 was set as the threshold.

## 3 Reagents and materials

### 3.1 Reagents

Serum creatinine (Scr, Cat. No. 20150526), blood urea nitrogen (BUN, Cat. No. 20150511), and hematoxylin-eosin stain (Cat. No. D006-1-1) were obtained from the Nanjing Jiancheng Institute of Biotechnology (Nanjing, China). Trizol reagent (Cat. No. F919KB3054) and a total RNA Isolation Kit (Cat. No. B511321) were purchased from Sangon Biotech (Shanghai, China). TB Green Premix Ex Taq (Cat. No. RR420A) and PrimeScript RT Reagent Kit with gDNA Eraser (Cat. No. RR047A) were purchased from Takara Biotechnology (Beijing, China). An Immunohistochemical Staining Kit (Lot No.16E06K04C1627) was purchased from Boster Biological Technology (Wuhan, China). Anti-c-Fos antibody (No. ab222699) and anti-JNK1 antibody (No. ab199380) were purchased from Abcam (Cambridge, United Kingdom). The primary antibodies against a-SMA (No. 19245), p-c-Fos (No. 5348), p-SAPK/JNK (Thr183/Tyr185) (No. 4668), p44/42 MAPK (Erk1/2) (No. 4695), p-p44/42 MAPK (Erk1/2) (No. 4370), and GAPDH (No. 5174) were produced by Cell Signaling Technology (Danvers, MA, United States). Enzyme-linked immunosorbent assay (ELISA) kits of IL-6 (Cat. No. 88-7064-88), TNF alpha (Cat. No. 88-7324-88), and IL-1β (Cat. No. 88-7013-88) were purchased from Thermo-Fisher Scientific (MA, United States).

### 3.2 Animals

#### 3.2.1 Dynamic experiment

Fifty male C57BL/6 mice (8 weeks old, 25 ± 2 g) were purchased from Beijing Vital River Laboratory Animal [License No. SCXK (Jing) 2014-0001] and housed in the Shanghai Model Organisms Center [License No. SYXK (Shanghai) 2017-0012]. The environment was free of specific pathogens, and the average relative humidity and temperature were 55%–60% and 25 ± 1°C, respectively. A 12-h light–dark cycle was used. All experiments were approved by the Institutional Laboratory Animal Care and Use Committee (IACUC) of the Shanghai Model Organisms Center (Approval No. IACUC 2018-0026).

#### 3.2.2 Pharmacodynamic experiment

Fifty C57BL/6 mice (8 weeks old, half male and half female, 25 ± 2 g) were purchased from Beijing Vital River Laboratory Animal [License No. SCXK (Jing) 2014-0001] and housed in the Laboratory Animal Center at the Shanghai University of Traditional Chinese Medicine [No. SYXK (Shanghai) 2020-0009]. All experiments were in accordance with the requirements of the Animal Ethics Committee of the Shanghai University of Traditional Chinese Medicine. The ethics committee registration number was PZSHUTCM200821017.

### 3.3 Chemicals and drugs

Aristolochic acid I (AAI) with the molecular formula of C_17_H_11_NO_7_ was purchased from Shanghai Standard Technology. The purity of AAI was 98.5% (Batch No. 3503). Sodium carboxymethyl cellulose (Batch No. F20051103) was obtained from Sinopharm Chemical Reagent Shanghai. FZHY extract powder was provided from Sundise Medicine Technology (SFDA approval No. Z20050546; Batch No. 20190608, the extract yield was 14.28% from Fuzheng Huayu formula). NAC (tradename: Flumucil) was obtained from Hainan Zambon Pharmaceutical (Cat. No. 1001542).

### 3.4 Animal groups and experimental design

#### 3.4.1 Dynamic experiment

Mice were randomly divided into a normal group (*n* = 12), 4-week model group (*n* = 12), 8-week model group (*n* = 12), and 12-week model group (*n* = 14). To prepare the AAI-induced renal fibrosis model, mice in model groups were intraperitoneally injected with 4 mg/kg AAI dissolved in 0.5% sodium carboxymethyl cellulose at a concentration of 1 mg/ml twice weekly for 12 weeks. Normal mice received 0.5% sodium carboxymethyl cellulose only. Mice were euthanized at 4 weeks, 8 weeks, and 12 weeks after AAI administration, respectively. The renal tissue and blood samples were collected. The blood was centrifuged at 3,000 rpm for 15 min to obtain the serum, and the kidneys were harvested and stored at −80°C or fixed in 4% paraformaldehyde solution.

#### 3.4.2 Pharmacodynamic experiment

Mice were randomly divided into a normal group (*n* = 10), model group (*n* = 10), FZHY low-dose (FL) group (*n* = 10), FZHY high-dose (FH) group (*n* = 10), and NAC group (*n* = 10). Preparation for the renal fibrosis model was the same as that for the dynamic experiment. Beginning from the fourth week of AAI injection, mice in the FL and FH groups were intragastrically administered FZHY at doses of 1.44 and 2.88 g/kg per day for 8 weeks, respectively. Mice in the NAC group was administered NAC at a dose of 3.0 g/kg per day for 8 weeks. Mice in the normal and AAI model groups were treated with an equal amount of vehicle. Mice were euthanized 12 weeks after AAI administration for renal tissue and blood sampling. Blood and kidney tissue sample processing was consistent with that described in the dynamic experiment section.

### 3.5 Biochemical assays in serum

Serum levels of Scr and BUN in mice were measured using the BIO-TEK multifunctional microplate reader (Molecular Devices, Sunnyvale, CA, United States) in accordance with the manufacturer’s instructions.

### 3.6 Hematoxylin-eosin and Sirius Red staining

Kidney tissue was cut along the horizontal plane. Half of the tissue was fixed with 4% formaldehyde for 48 h, dehydrated using an automated dehydrator, and embedded in paraffin. The tissue was then cut into 4-μm paraffin sections for hematoxylin-eosin staining to assess renal injury and for Sirius Red staining to assess collagen deposition.

### 3.7 Quantitative reverse transcription-polymerase chain reaction

Based on the results of the network pharmacology, the top 10 potential hub genes were selected for quantitative reverse transcription-polymerase chain reaction (qRT-PCR) verification and screening. The National Center for Biotechnology Information online primer design software Primer-Blast was used to design primers (*INS2*, *IL-6*, *VEGFA*, *CASP3*, *MAPK8*, *MYC*, *ESR1*, *FOS*, *CCND1*, and *β-actin*), which were synthesized by Shanghai Sangon Biotech. *EGFR* primer sequences were provided by BIOTNT (see Table 1 for gene names and primer sequences). Total RNA was extracted from mice renal tissue by using Trizol reagent. Reverse transcription was performed using a reverse transcription kit, and cDNA amplification was performed using an SYBR Premix Ex Taq II fluorescent quantitative kit. Each sample was tested three times. The 2^-△△CT^ method was used for analysis and quantification, and *β-actin* was the internal reference.

**TABLE 1:** qRT-PCR primer sequences of each gene.

Gene	Forward Primer(5′-3′)	Reverse Primer(5′-3′)
INS	CCATCAGCAAGCAGGAAGGTA	GGCTGGGTTGAGGATAGCAAA
IL6	TAGTCCTTCCTACCCCAATTTCC	TTGGTCCTTAGCCACTCCTTC
VEGFA	GGCTGCTGTAACGATGAA	CTGCTGTGCTGTAGGAAG
EGFR	AACAGGCTTTTTGCTGATTCA	TGACCATGTTGCTTTGTTCTG
CASP3	AGGCAAACACTGAAGGCAGA	TAGCCAGGTTTAGGGACACC
MAPK8	GAGGAACGAACTAAGAATGGA	ATTGACAGACGGCGAAGA
MYC	TGTAGTAATTCCAGCGAGAG	CAGATTGTAAGTTCCAGTGAG
ESR1	TGCTCCTAACTTGCTCCT	GATGTGGTCCTTCTCTTCC
FOS	TGAAGACCGTGTCAGGAG	CGCTTGGAGTGTATCTGTC
CCND1	CAGAAGTGCGAAGAGGAG	GGATAGAGTTGTCAGTGTAGA
*β-actin*	TGACGAGGCCCAGAGCAAGA	ATGGGCACAGTGTGGGTGAC

### 3.8 Immunohistochemistry staining

The tissue sections were dewaxed and hydrated in graded ethanol and microwaved in sodium citrate buffer to repair antigens. Endogenous peroxidase activity was reduced using 3% H_2_O_2_ for 10 min. Each sample was blocked with 5% bovine serum albumin for 20–30 min and then incubated with the primary antibody against a-SMA (1:400), Fos (1:2000) at 4°C overnight. On the next day, the sections were incubated with a biotin-labeled goat antirabbit secondary antibody for 1 h and then stained with 3,3-diaminobenzidine and counterstained with hematoxylin. After dehydration and drying, the sections were mounted with neutral gum and observed under a microscope. Aperio Image Scope software was used to perform image analysis.

### 3.9 Western blot analysis

Kidney tissues were homogenized in RIPA lysis buffer [50 mM Tris-HCl (pH 7.4), 150 mM NaCl, 0.1% sodium dodecyl sulfate (SDS), 1% Nonidet P-40, 1 mM EDTA, 1 mM PMSF, and 1 × Roche Complete Mini protease inhibitor cocktail]. The supernatants were collected after centrifugation at 10 ,000 × *g* at 4°C for 15 min. The protein concentration was determined using a bicinchonic acid protein assay kit. An equal amount of protein was separated through 10% SDS gel electrophoresis under denaturing conditions and was then transferred to the nitrocellulose membrane. The membrane was blocked with 5% nonfat milk in TBST at room temperature for 1 h and incubated with a primary antibody [a-SMA, 1:1000; c-Fos, 1:1000; JNK1, 1:1000; p-c-Fos, 1:1000; p-JNK, 1:1000; p44/42 MAPK (Erk1/2), 1:1000; p-p44/42 MAPK (p-Erk1/2), 1:2000] or GAPDH (1:2000) at 4°C overnight. After being washed in TBST, the blots were incubated with a horseradish-conjugated secondary antibody (No.8884). The signals were visualized using the Odyssey infrared laser imaging system by obtaining with photographs of bands, and ImageJ software was used to analyze the grey value.

### 3.10 Enzyme-linked immunosorbent assay

The concentrations of *IL-1*β, *IL-6*, and *TNF-*α in kidney tissues were determined using an enzyme-linked immunosorbent assay (ELISA) kit. Kidney homogenates were obtained after homogenizing in 50 mM PBS buffer (pH 7.4) in an ice water bath and centrifuging at 10 ,000 × *g* for 15 min at 4°C. For each assay, 100 µl of kidney homogenates from all mice were serially diluted to ensure that values obtained were within the linear range of the standards provided with each kit. The manufacturer’s instructions (Thermo-Fisher Scientific) were strictly followed during the ELISA experiments.

### 3.11 Statistical analysis

The experimental data were statistically analyzed using SPSS 26.0 and were represented as the mean ± standard deviation (SD). One-way analysis of variance was used to compare groups, and *p* < 0.05 indicated a statistically significant difference. GraphPad Prism eight software was used for mapping.

## 4 Results

### 4.1 Prediction of chronic kidney disease-Related genes from Gene Expression Omnibus profiles

**FIGURE 1:**
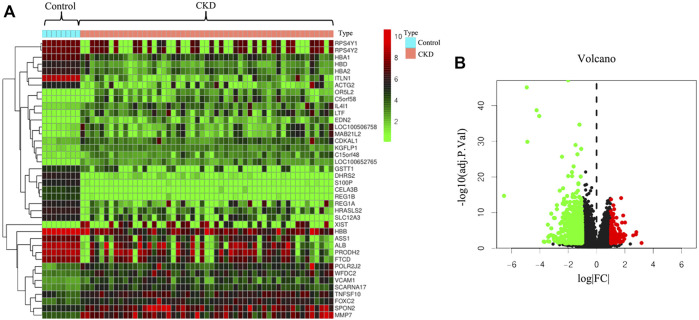
Prediction of differentially expressed genes between patients with chronic kidney disease (CKD) and healthy donors. **(A)** Heatmap of differentially expressed genes in human kidney tissues with or without CKD. **(B)** Volcano plot of distribution of differentially expressed genes.

To further illustrate the pathological mechanisms of CKD in humans, DEGs between healthy donors and patients with CKD were screened (Figure 1). In total, 815 genes in the kidney biopsy samples were expressed among patients with CKD and not among those without CKD. Of the 815 genes, 129 genes were upregulated, and 686 genes were downregulated. The heatmap (Figure 1A) shows the top 40 DEGs in the two groups. Patients with CKD expressed the following genes which different from the individuals without CKD: *RPS4Y1*, *RPS4Y2*, *HBA1*, *HBD*, *HBA2*, *ITLN1*, *ACTG2*, *OR5L2*, *C5orf58*, and *IL4I1*. The volcano plot (Figure 1B) was applied to demonstrate the distribution of gene expression differences; the horizontal axis is the logarithm of the fold differences, and the vertical axis is the negative logarithm of the *p*-value for multiple significant differences. Red dots represent significantly upregulated genes, and green dots represent significantly downregulated genes (*p* < 0.05 and log |FC| ≥1.2).

### 4.2 Potential hub genes through which Fuzheng Huayu acts on chronic kidney disease

**FIGURE 2:**
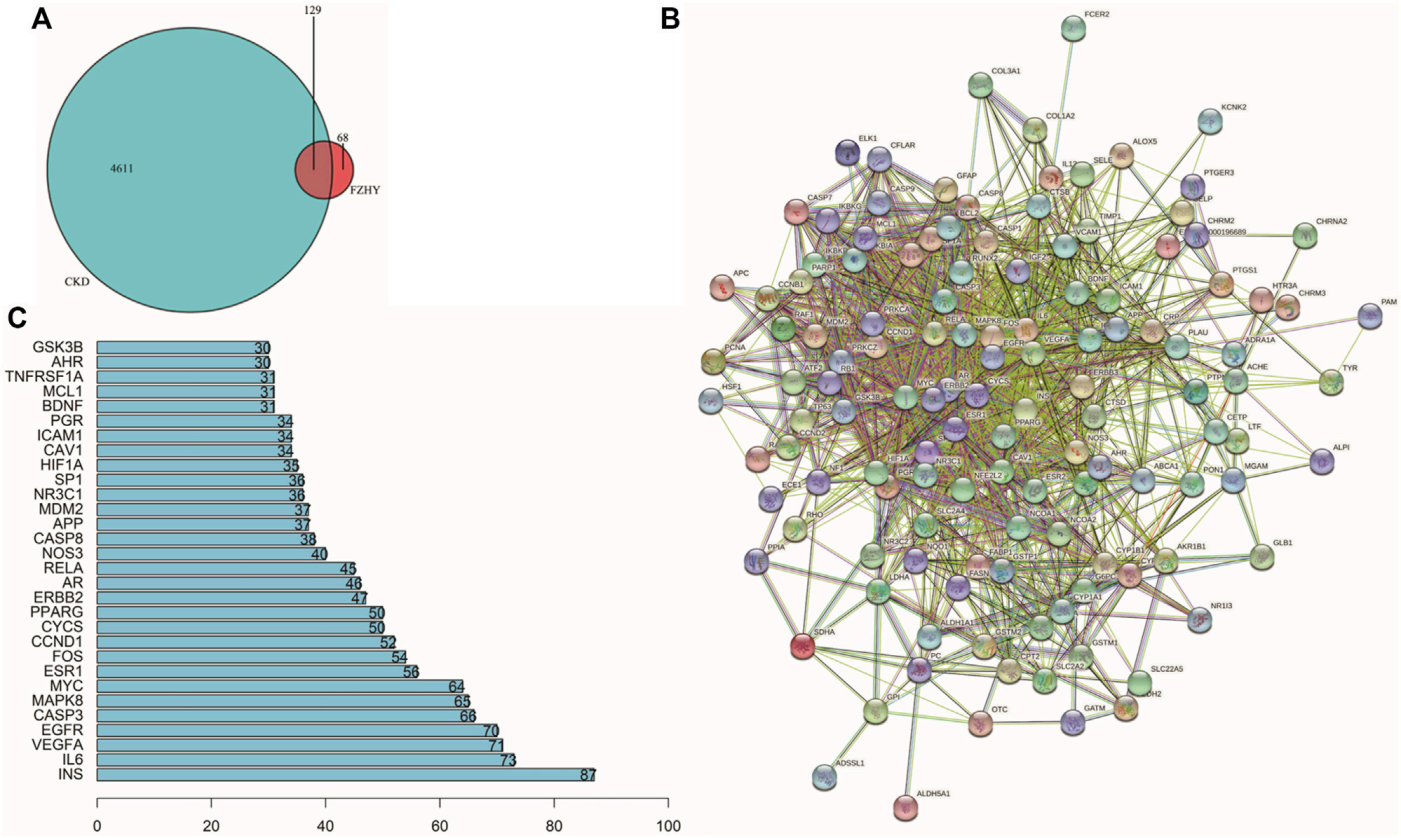
Construction of PPI network. **(A)** Venn diagram of 129 target genes of Fuzheng Huayu (FZHY) and chronic kidney disease (CKD). Overlaps are considered potential target genes of FZHY acting on CKD. **(B)** Protein–protein interaction network constructed using STRING. **(C)** Bar plot of potential hub proteins. *Y*-axis represents name of the protein. *X*-axis represents number of adjacent proteins. Height denotes the number of protein connections.

A Venn diagram (Figure 2A) was drawn to illustrate the common genes between FZHY and CKD. In total, 129 genes were potential hub genes responsible for the effects of FZHY on CKD. To further illustrate the relationship between FZHY and CKD target proteins, PPI network (Figure 2B) was constructed using the Search Tool for the Retrieval of Interacting Genes/Proteins public database. The bar plot (Figure 2C) was used to display the top 30 potential hub genes through which FZHY acts on CKD, such as *INS*, *IL-6*, *VEGFA*, *EGFR*, *CASP3*, *MAPK8*, and *MYC*.

### 4.3 Gene ontology and Kyoto Encyclopedia of Genes and Genomes pathway analysis

**FIGURE 3:**
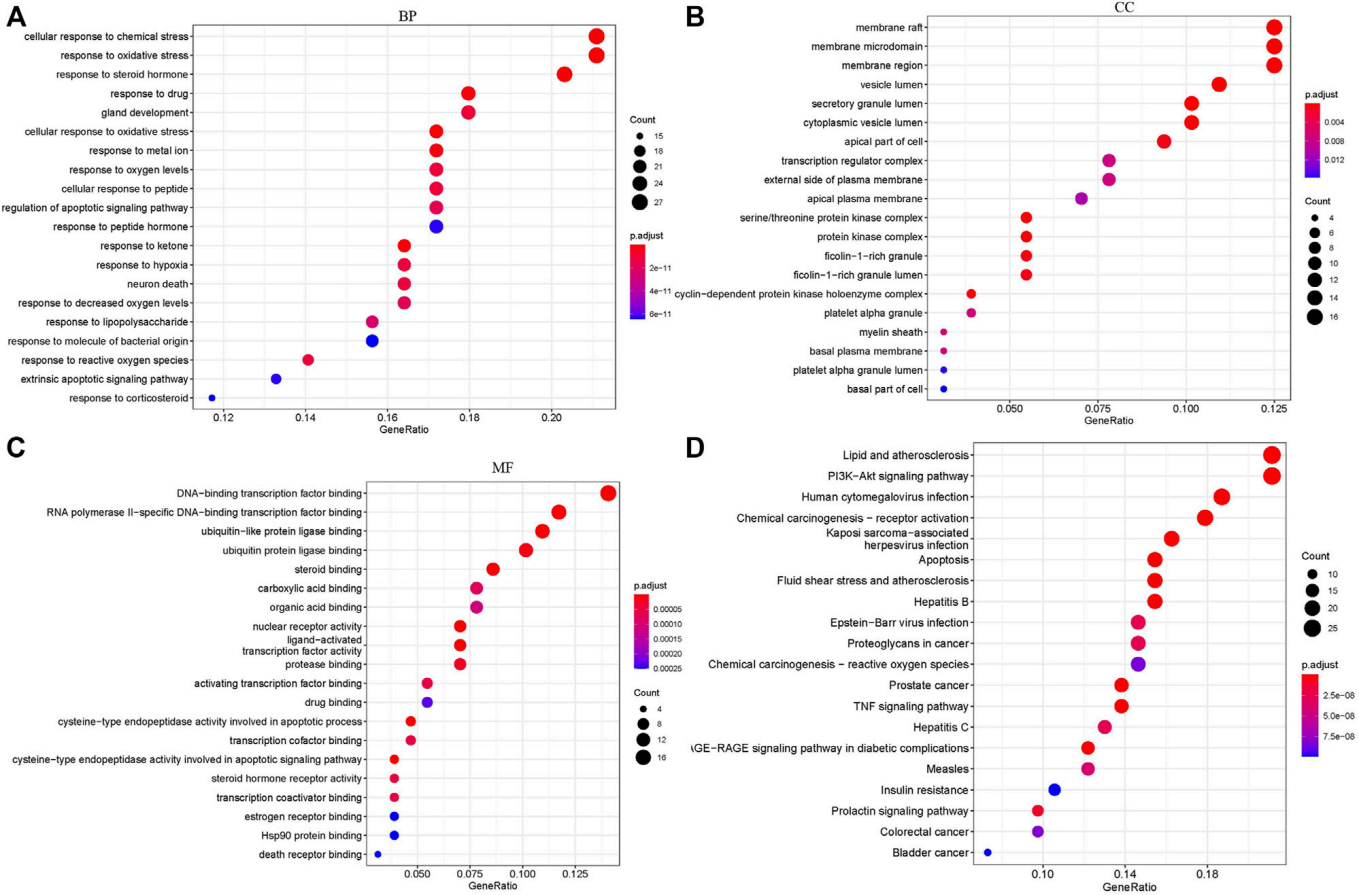
Target biological function analysis: GO and KEGG analysis. **(A)** BP analysis of 129 FZHY–CKD target genes. **(B)** CC analysis of 129 FZHY–CKD target genes. **(C)** MF analysis of 129 FZHY–CKD target genes. **(D)** KEGG pathway enrichment analysis of 129 FZHY–CKD target genes. KEGG, Kyoto Encyclopedia of Genes and Genomes; BP, biological process; MF, molecular function; CC, cellular component.

GO annotations of the 129 potential therapeutic target genes were performed to identify the biological activity of FZHY against renal fibrosis. The top 20 significantly enriched terms in the categories of BP, CC, and MF are shown in Figure 3. The BP (Figure 3A) was mainly related to the cellular response to chemical stress, response to steroid hormone production, response to ketone, and response to oxidative stress. The CC (Figure 3B) was related to the membrane raft, membrane microdomain, membrane region, vesicle lumen, and secretory granule lumen. The MF (Figure 3C) was related to DNA-binding transcription factor binding, nuclear receptor activity, ligand-activated transcription factor activity, and steroid binding. Furthermore, the KEGG pathway analysis (Figure 3D) indicated that the lipid and atherosclerosis pathways, prostate cancer pathway, apoptosis pathway, fluid shear stress and atherosclerosis pathways, and TNF signaling pathway may be correlated with the therapeutic effects of FZHY on renal fibrosis.

### 4.4 Effect of Fuzheng Huayu on renal function and fibrosis caused by Aristolochic acid I

**FIGURE 4:**
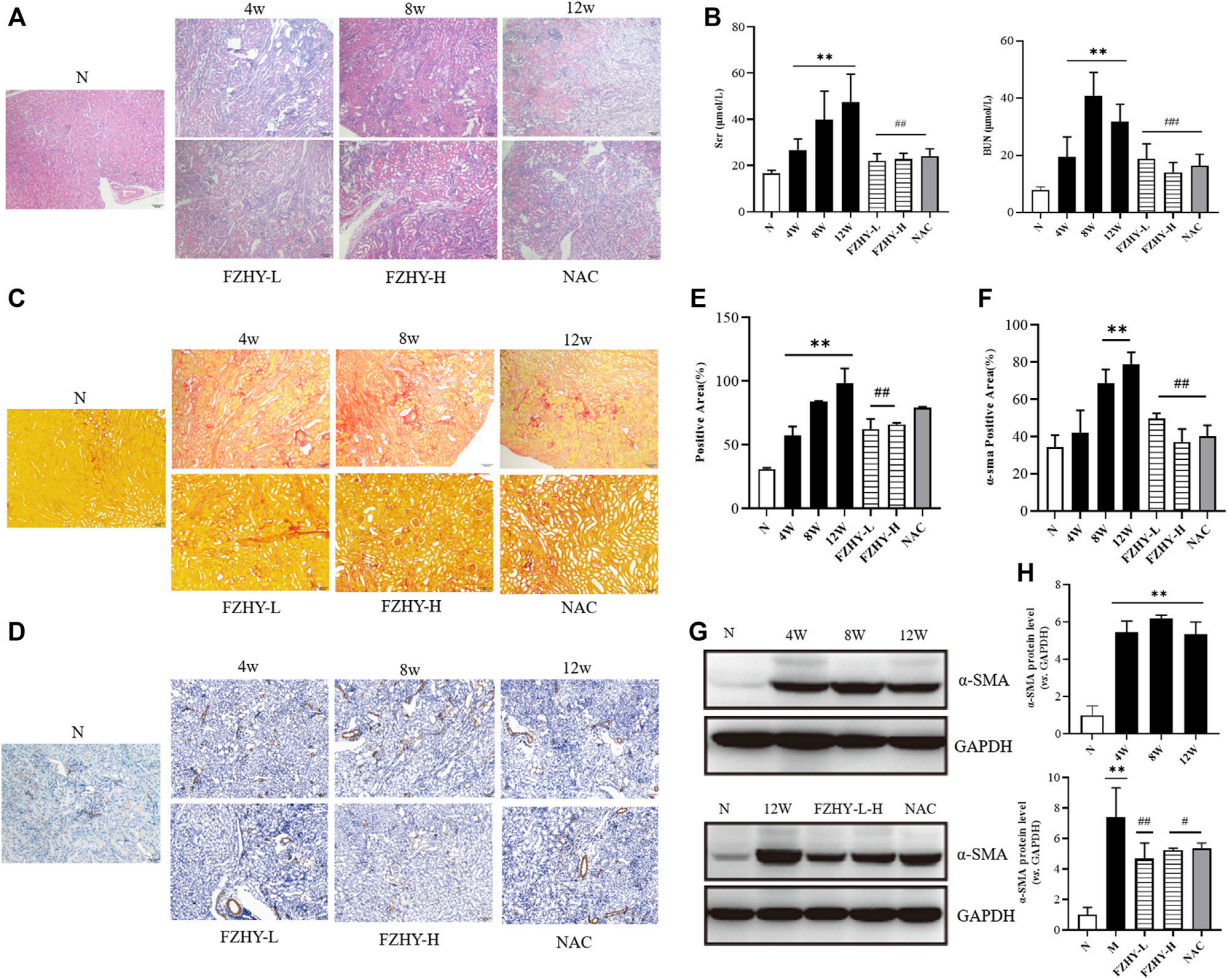
Fuzheng Huayu (FZHY) alleviates renal fibrosis induced by Aristolochic Acid I (AAI) *in vivo*. Dynamic experimental model mice were intraperitoneal injected with AAI (4 mg/kg body weight) twice a week and sacrificed at 4 weeks, 8 weeks, and 12 weeks. Pharmacodynamic experimental model mice were intraperitoneal injected with AAI (4 mg/kg body weight) twice a week for 12 weeks. From fourth week of AAI injection, mice were intragastrically administered FZHY (1.44 g/kg, 2.88 g/kg body weight) or NAC (3.0 g/kg body weight) daily for 8 weeks. All mice were sacrificed. Hematoxylin-eosin **(A)** staining of mouse renal tissue sections demonstrated degree of renal injury. Levels of serum creatinine and blood urea nitrogen **(B)** were used to detect renal function. The collagen deposition was shown by using Sirius Red staining **(C)**. Significant renal collagen deposition was observed in mice after AAI administration, which was then reduced by using FZHY or NAC **(E)**. IHC **(D,F)** and WB **(G,H)** analysis of a-SMA showed the degree of renal fibrosis, which increased after AAI injection and alleviated by FZHY (Figures 4F,H). FL, Fuzheng Huayu low-dose; FH, Fuzheng Huayu high-dose. Bar = 100 μm; ^*^ *p* < 0.05, compared with normal group; ^**^ *p* < 0.01, compared with normal group; ^#^ *p* < 0.05, compared with model group; ^##^ *p* < 0.01, compared with model group.

The dynamic and pharmacodynamic AAI mice models were used to verify the network pharmacology results. The hematoxylin-eosin staining results (Figure 4A) revealed that the renal tissue structure of the normal group was intact, and no obvious abnormality nor inflammatory cell infiltration was presented in the renal interstitium. However, renal tissue structure destruction was evident in the model group. Tubular dilatation and edema indicated clear interstitial inflammatory cell infiltration. The basement membrane of the renal tubules was incomplete, and the epithelial cells of the proximal convoluted tubule were sloughed off when the tubulointerstitium widened. Compared with the model group, the FZHY group exhibited reduced inflammatory cell infiltration, along with the alleviation of the damage to renal tubules. As shown in Figure 4B, after AAI injection, the Scr and BUN levels increased gradually over time and were significantly higher at 8 weeks and 12 weeks, respectively (*p* < 0.01), compared with the normal group (Figure 4B). After the use of FZHY, the Scr and BUN levels significantly decreased compared with the 12 weeks model group (*p* < 0.01). Sirius Red staining (Figure 4C) demonstrated the collagen deposition. As the result, after AAI administration, significant renal collagen deposition was observed in mice, which was reduced by using FZHY or NAC (Figure 4E). IHC (Figure 4D) and WB (Figure 4G) analysis of a-SMA were used to demonstrate the degree of renal fibrosis in mice. Compared with the normal group, the degree of renal fibrosis was increased after AAI injection, and alleviated by FZHY(Figures 4F,H).

**FIGURE 5:**
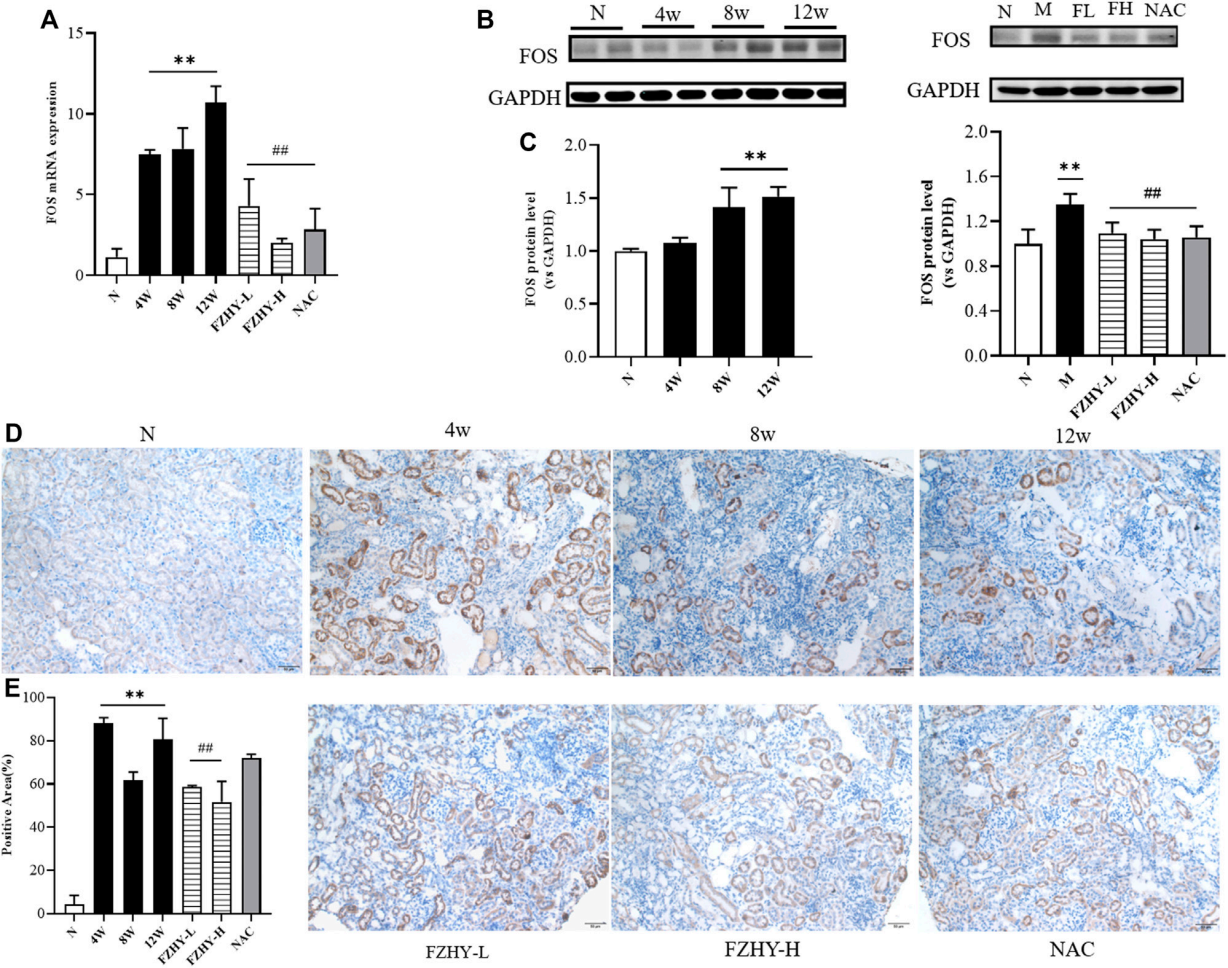
*FOS* may serve as key gene for FZHY to act on chronic kidney disease. Dynamic experimental model mice were intraperitoneal injected with Aristolochic Acid I (AAI) (4 mg/kg body weight) twice a week and sacrificed at 4 weeks, 8 weeks, and 12 weeks, respectively. Pharmacodynamic experimental model mice were intraperitoneal injected with AAI (4 mg/kg body weight) twice a week for 12 weeks. From fourth week of AAI injection, mice were intragastrically administered FZHY (1.44 g/kg, 2.88 g/kg body weight) or NAC (3.0 g/kg body weight) per day for 8 weeks. All mice were sacrificed. Quantitative real-time polymerase chain reaction analysis of mice renal tissues **(A)** shows that *FOS* gene expression significantly increased at 4 weeks, 8 weeks, and 12 weeks compared with normal group and decreased after use of FZHY. Western blot exhibited **(B)** *FOS* protein expression in mice renal tissue. *FOS* protein significantly increased at 8 weeks and 12 weeks compared with normal group and decreased after a high dose of FZHY or NAC **(C)**. Immunohistochemistry was used for locating *FOS* protein **(D)** and quantifying **(E)** in paraffin sections of mice renal tissue. *FOS* protein is mainly expressed in tubular epithelial cells, mostly in cytoplasm, and some are expressed in nucleus. Bar = 50 μm; ^*^ *p* < 0.05, compared with normal group; ^**^ *p* < 0.01, compared with normal group; ^#^ *p* < 0.05, compared with model group; ^##^ *p* < 0.01, compared with model group.

Based on the results of the network pharmacology, the top 10 genes were selected to verify their reliability through qRT-PCR. Analysis of the renal tissues of the AAI dynamic mice model revealed that among the top 10 potential hub genes, the expression of *INS*, *EGFR*, *IL-6*, *CASP3*, *MAPK8*, *FOS*, *MYC*, *CCND*, and *VEGFA* was higher than that in the normal group. Of these, the differential expression was most pronounced with the *FOS* gene. Especially, the amount of mouse *FOS* gene expression in the 12w model group was about 10 times of that in the normal group (*p* < 0.01). (Figure 5A).

According to the literature, *FOS* is a downstream transcription factor, which can form AP-1 with *JUN*. We selected the *FOS* gene for subsequent studies as it plays the crucial role in regulating cell proliferation and death (Shaulian & Karin, 2002). In the mouse pharmacodynamic model of AAI, *FOS* gene expression decreased in the FZHY group and the positive drug NAC group compared with the 12-week model group (*p* < 0.01) (Figure 5A). Consistent with the qRT-PCR results, the Western blot analysis results demonstrated that *FOS* may be the target gene for the FZHY decoction against renal fibrosis (Figures 5B,C). According to the immunohistochemistry results, *FOS* protein mainly located in the renal tubular epithelial cells, most of which are present in the cytoplasm and only a few are located in the nucleus (Figures 5D,E). These results revealed that *FOS* is mainly expressed in tubular epithelial cells and serves as a potential target gene against CKD.

### 4.5 Role of mitogen-activated protein kinase family/FOS signaling pathways in Fuzheng Huayu against renal fibrosis

As one of the immediate early response genes, *FOS* expression is regulated by several extracellular signaling molecules, including those involved in the MAPK signaling pathways (mainly JNK and ERK signaling pathways) (Dalhäusser et al., 2022). Based on the GO annotation and KEGG analysis data, we selected p-c-FOS, JNK, p-JNK, ERK, and p-ERK for further validation.

**FIGURE 6:**
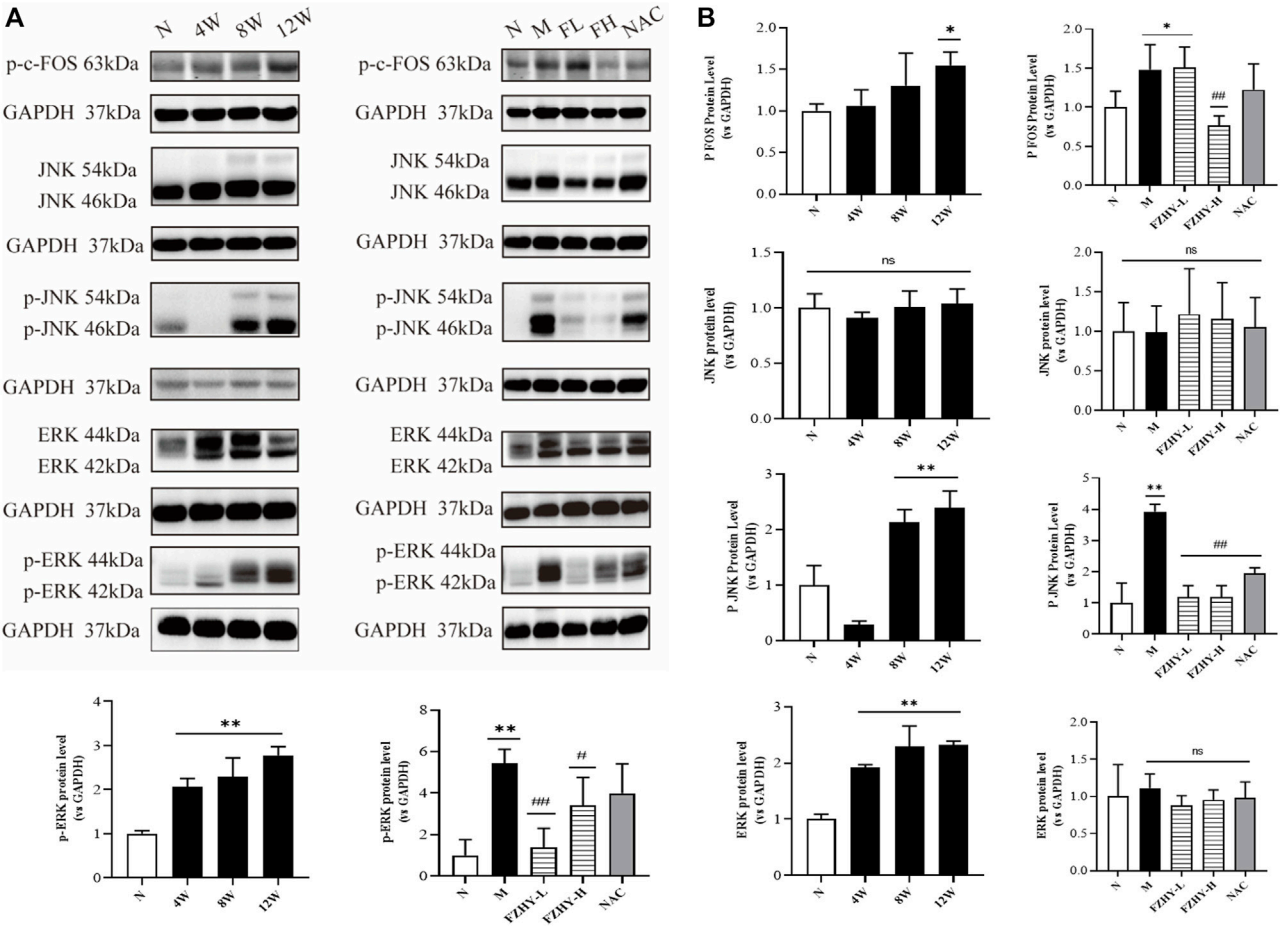
Western blot analyses further illustrate the mechanisms of FZHY against renal fibrosis. Dynamic experimental model mice were intraperitoneal injected with Aristolochic Acid I (AAI) (4 mg/kg body weight) twice a week and sacrificed at 4 weeks, 8 weeks, and 12 weeks. Pharmacodynamic experimental model mice were intraperitoneal injected with AAI (4 mg/kg body weight) twice a week for 12 weeks. From the fourth week of AAI injection, mice were intragastrically administered FZHY (1.44 g/kg, 2.88 g/kg body weight) or NAC (3.0 g/kg body weight) per day for 8 weeks. All mice were sacrificed. p-c-fos, JNK, p-JNK, ERK, and p-ERK expression in mice renal tissues were analyzed using Western blot analysis **(A)**. p-JNK, ERK, and p-ERK significantly increased at both 8 weeks and 12 weeks after AAI administration **(B)**. After the FZHY gavage, protein expression levels of JNK and ERK did not change significantly, but expression levels of p-JNK and p-ERK significantly decreased compared with model group **(B)**. ^*^ *p* < 0.05, compared with normal group; ^**^ *p* < 0.01, compared with normal group; ^#^ *p* < 0.05, compared with model group; ^##^ *p* < 0.01, compared with model group; ns, no significance, compared with model group.

As shown in Figures 6A,B, p-c-FOS, p-JNK, ERK, and p-ERK expression significantly increased at both 8 weeks and 12 weeks after AAI administration. After FZHY gavage, the expression levels of JNK and ERK proteins did not significantly change, and the expression levels of p-c-FOS p-JNK, and p-ERK significantly decreased compared with those in the model group (*p* < 0.01). The results suggest that the protective effect of FZHY on renal fibrosis may be related to the inhibition of the stimulation of the MAPK signaling pathways.

**FIGURE 7:**
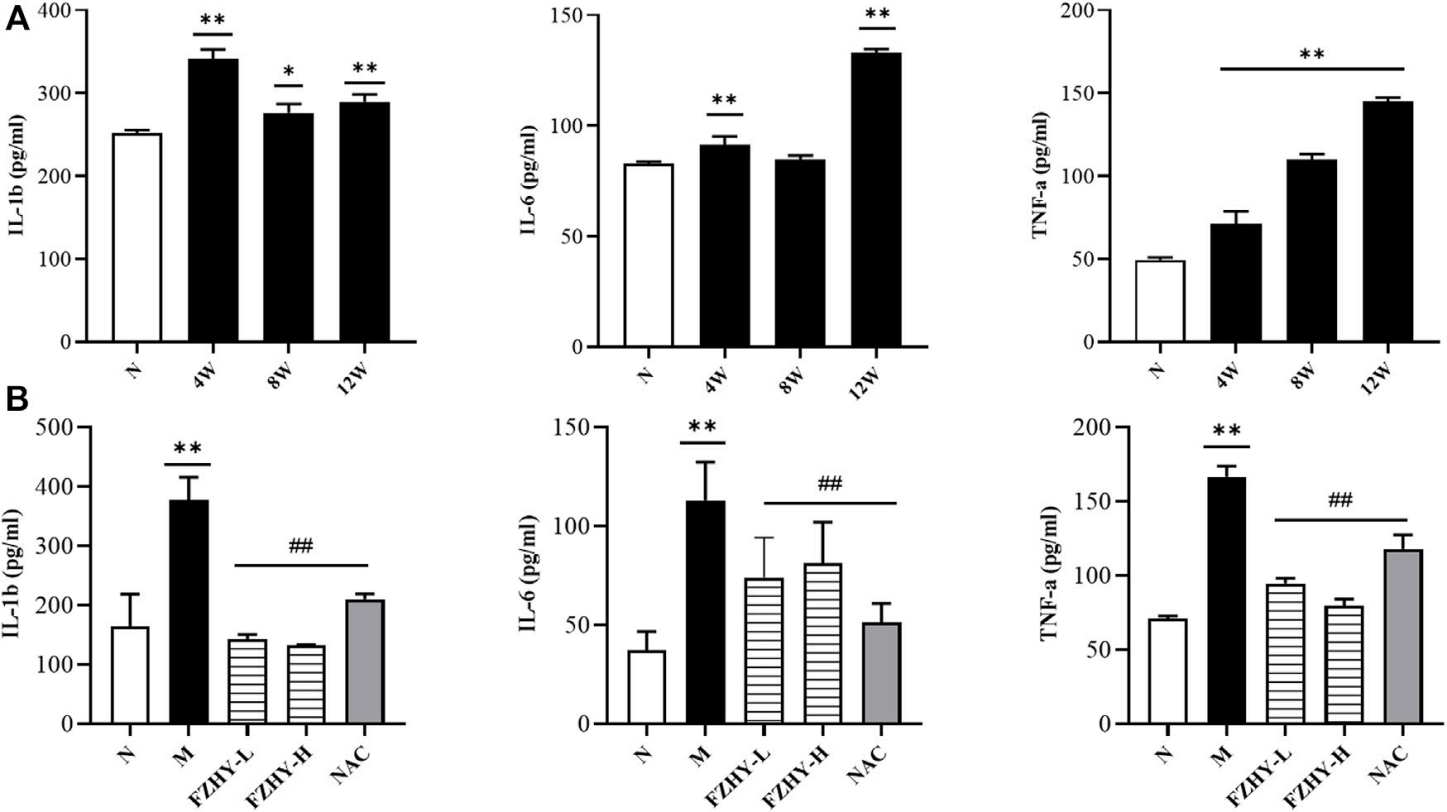
Enzyme-linked immunosorbent assay (ELISA) analyses demonstrate the changes of inflammatory response during FZHY against renal fibrosis. Dynamic experimental model mice were intraperitoneal injected with Aristolochic Acid I (AAI) (4 mg/kg body weight) twice a week and sacrificed at 4 weeks, 8 weeks, and 12 weeks. Pharmacodynamic experimental model mice were intraperitoneal injected with AAI (4 mg/kg body weight) twice a week for 12 weeks. From the fourth week of AAI injection, mice were intragastrically administered FZHY (1.44 g/kg, 2.88 g/kg body weight) or NAC (3.0 g/kg body weight) per day for 8 weeks. All mice were sacrificed. ELISA analysis was employed to detect inflammatory factors. Levels of *IL-6*, *IL-1β*, and *TNF-α* significantly increased after AAI administration **(A)**, and levels of *IL-6*, *IL-1β*, and *TNF-α* decreased after FZHY **(B)**.

Because the activation of the MAPK signaling pathways was correlated with inflammatory factors, ELISA was conducted (Figures 7A,B). The levels of *IL-6*, *IL-1β*, and *TNF-α* significantly increased after AAI administration, and the levels of *IL-6*, *IL-1β*, and *TNF-α* decreased after FZHY administration, suggesting that FZHY alleviates the inflammatory response induced by AAI.

**FIGURE 8:**
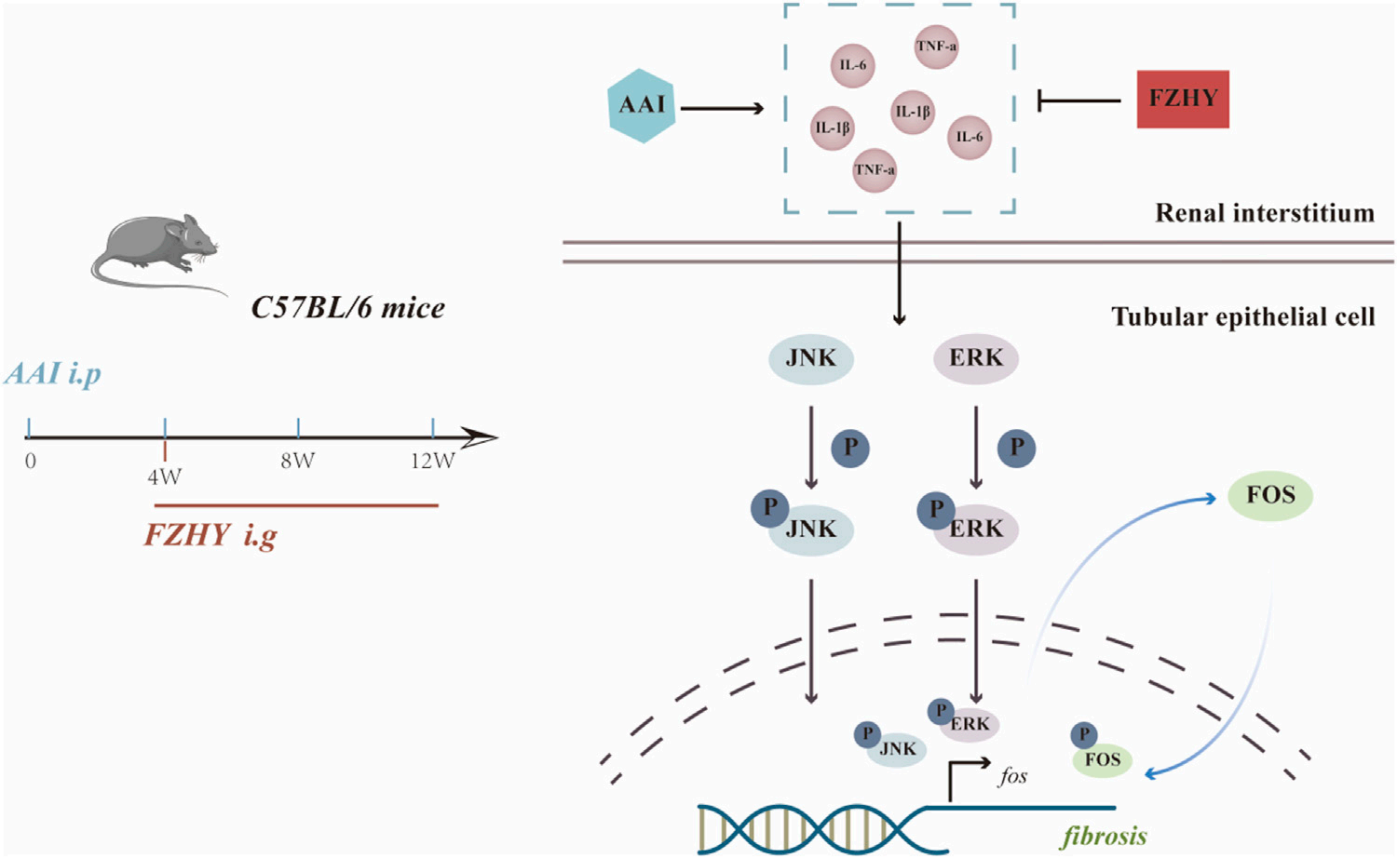
Diagram depicting the potential mechanism of FZHY against the renal fibrosis induced by AAI.

Generally, after the stimulation of MAPKs by inflammatory factors, the phosphorylated JNK, ERK proteins enter the nucleus and promote the transcription of the *c-fos* gene, which in turn can further promote fibrosis. Our research shows that FZHY could alleviate AAI-induced renal injuries and inflammatory responses through the MAPK/FOS signaling pathways (Figure 8).

## 5 Discussion

CKD has the characteristics of high incidence, high prevalence, and complex mechanisms. End-stage kidney disease caused by CKD affects countless people worldwide. According to statistics, approximately 10% of adults were affected by CKD (GBD Chronic Kidney Disease Collaboration., 2020). Concomitant with high prevalence, CKD also has a high mortality rate. According to statistical studies, the number of deaths due to CKD is expected to reach 2.2 to 4 million by 2040 (Foreman et al., 2018). As the hallmark of progressive CKD, renal fibrosis is mainly characterized by glomerulosclerosis, tubular atrophy, and renal interstitial fibrosis (Yan et al., 2021). Renal fibrosis is the final pathway in virtually all chronic progressive kidney diseases, regardless of initial etiology (Humphreys., 2018). Renal fibrosis has a complex pathogenesis involving multiple signaling pathways, such as the TGF-β, angiotensin II, and Wnt signaling pathways (Yuan et al., 2022). Although drugs targeting the inflammatory factors associated with signaling pathways have been approved for testing in clinical trials, no major advances have been made specifically in treatment strategies for renal fibrosis (Prakoura et al., 2019).

In the last decades, TCM has attracted worldwide attention for its curative effects, relatively low toxicity, and low cost (Luo et al., 2020). As a clinical prescription, FZHY has been shown to improve liver function, reduce Child Pugh scores, and alleviate symptoms in clinical studies (Chen et al., 2019). Multicenter phase II clinical trials in the United States have revealed that FZHY has a positive effect on hepatitis C liver fibrosis (Hassanein et al., 2022). Our previous studies have indicated that FZHY can effectively treat HgCl_2_-induced renal interstitial fibrosis associated with *TGF-β1*-induced epithelial-to-mesenchymal transition (Wang et al., 2010) and peroxidation (Yuan et al., 2017). A randomized, double-blind, multicenter clinical trial was conducted to evaluate the efficacy of FZHY for CKD (2019ZX09201001-001-006). TCM has multitarget characteristics. Therefore, exploring the mechanisms of FZHY against renal fibrosis is essential to understand how to best use FZHY.

In this study, we identified 815 genes that were differentially expressed in patients with CKD compared with normal individuals in their kidney tissues, of which 129 genes were upregulated and 686 genes were downregulated (Figure 1). Then, after searching the public database, the target network of FZHY against CKD was constructed and analyzed. The Venn diagram indicated 129 potential genes. The PPI network of FZHY–CKD was used to explore the interaction between the formula and disease. GO annotations and the KEGG pathway were used for the functional analysis of related target genes. We identified 1753 GO terms that were significantly associated with FZHY treatment (Figure 3). Several terms were also associated with biological processes relevant to renal diseases, including the response to chemical stress (Kitamura., 2008), response to oxidative stress (Aranda-Rivera et al., 2021), response to drugs (Cybulsky., 2017), and epithelial cell proliferation (Bonventre., 2014). These terms may be associated with the ability of FZHY to inhibit renal fibrosis.

Our KEGG pathway analyses also revealed 128 pathways that were differentially regulated in response to FZHY treatment (Figure 3), including the PI3K–Akt signaling pathway, MAPK signaling pathways (Yuan et al., 2022), and TNF signaling pathway (Al-Lamki & Mayadas., 2015), all of which are related to the development of renal fibrosis. These results jointly indicate that FZHY can reverse renal fibrosis by regulating the aforementioned signaling pathways, although additional research is required to validate these findings. The bar plot presents the top 30 potential hub genes through which FZHY acts on CKD, such as *IL-6* (Feigerlová & Battaglia-Hsu., 2017), *EGFR* (Rayego-Mateos et al., 2018), *MAPK8*, and *FOS* (Grynberg et al., 2017). These proteins are also associated with the development of renal disease.

CKD can be caused by various factors, including the inappropriate use of drugs (Romagnani et al., 2017) and prolonged exposure to a toxicant–contaminated environment (i.e., Balkan endemic nephropathy due to AAI) (Lukinich-Gruia et al., 2022). Aristolochic acids (AAs), which are produced by *Aristolochia* and *Asarum*, have been widely used as herbal TCMs (Anger et al., 2020). AAⅠ and AAⅡ are active components in AAs (Jadot et al., 2017). AA nephropathy (AAN) is clinically characterized by rapidly progressive interstitial fibrosis, including impaired proximal tubular function (Luciano & Perazella, 2015). The mechanisms of AAI-induced kidney disease include oxidative stress, apoptosis, and an inflammatory response (Anger et al., 2020). Studies have demonstrated that AAs can cause transitional cell carcinoma of the renal pelvis, ureter, and bladder epithelium by binding to cellular DNA and forming AA–DNA adducts (Stiborová et al., 2017).

Currently, many Chinese herbal medicines containing AAI have been banned, but Aristolochiaceae plants are still applied in clinical treatment due to their relatively low AAI content. Based on the pathological characteristics and pathogenic mechanisms of AAN, AAI was used to establish the CKD mice model in this study.

The dynamic model of AAI at 12 weeks in mice and the pharmacodynamic model of AAI plus FZHY were used for network pharmacological validation. After AAI administration, the Scr and BUN levels of the model group were higher than those of the normal group, and the BUN level significantly decreased after using FZHY (Figure 4B). Hematoxylin-eosin staining revealed that in the AAI model, the renal tissue structure was significantly disrupted, with obvious tubular dilatation, interstitial inflammatory cell infiltration, epithelial cell sloughing, and atrophy in the proximal convoluted tubules. Compared with the model group, the FZHY group exhibited slightly reduced infiltration of inflammatory cells, and renal tubular damage was alleviated (Figure 4A). Moreover, the collagen deposition and the degree of fibrosis in the FZHY and NAC groups decreased compared with that in the model group (Figures 4C,D). Therefore, the results suggest that FZHY can improve, to some degree, renal interstitial fibrosis induced by AAI.

Based on the network pharmacological results, we selected the top 10 genes to perform the qRT-PCR and verify their reliability. Among the top 10 genes, the expression of *EGFR*, *INS*, *IL-6*, *CASP3*, *MAPK8*, *FOS*, *MYC*, *CCND*, and *VEGFA* increased compared with that in the normal group. *FOS* elevation was the most prominent and remained high at 12 weeks (Figure 5A). Immunohistochemistry indicated that *FOS* is mainly localized in tubular epithelial cells and expressed in the cytoplasm, with some located in the nucleus (Figure 5D). Similarly, the results of the Western blot analysis demonstrated that *FOS* may serve as a target gene through which FZHY exerts its effects against renal fibrosis (Figures 5B,C).

The proto-oncogene c-Fos is a gene coding for the 62-kDa protein comprising 380 amino acids (Alfonso-Gonzalez & Riesgo-Escovar, 2018). The c-Fos protein is a regulator of cell functions, such as differentiation, proliferation, and transformation (Tsiambas et al., 2020). Based on the characteristics of the *FOS* gene and the role of the MAPK signaling pathways in inflammation and injury (Bhosale et al., 2022), we performed a WB analysis of p-c-Fos, JNK, p-JNK, ERK, and p-ERK expression. p-JNK, ERK, and p-ERK expression significantly increased in the AAI model at 8 and 12 weeks (Figures 6A,B). However, the protein expression levels of JNK and ERK did not change significantly, and the expression levels of p-JNK and p-ERK significantly decreased compared with those in the model group (*p* < 0.01), suggesting that the protective effect of FZHY on the kidney may be related to the inhibition of the MAPK signaling pathways. Studies have shown that AAI can lead to renal inflammatory responses (Anger et al., 2020), such as the expression of the proinflammatory factors IL6, TNF-α (Li et al., 2021), IL-1β. These factors, in turn, can activate the MAPK signaling pathway (Gaestel et al., 2009; Garbers et al., 2018). ELISA results showed that FZHY could inhibit the inflammatory response caused by AAI (Figures 7A,B).

Collectively, FZHY could alleviate AAI-induced renal injuries and inflammatory responses by inhibiting the MAPK/FOS signaling pathways (Figure 8). Our study identified that *FOS* may play a vital role in how the FZHY formula acts on CKD, and the *MAPK* signaling pathway may serve as the key point. However, further research is necessary both *in vivo* and *in vitro*.

## 6 Conclusion

The mechanisms of the FZHY formula that alleviate renal fibrosis in CKD have been preliminarily explored using network pharmacological predictions and *in vivo* validation. Functional enrichment analyses demonstrated that FZHY may achieve therapeutic efficacy by modulating MAPK and PI3K-AKT signaling and reactive oxygen species. However, further research is necessary to validate the role of FZHY in functional contexts. Our work also revealed that FZHY can protect the kidney from inflammatory injuries caused by AAI and antagonize inflammatory factor-stimulated MAPK/FOS activation.

## Data Availability

The datasets presented in this study can be found in online repositories. The names of the repository/repositories and accession number(s) can be found in the article/Supplementary Material.

## Glossary

AAI: Aristolochic acid I
ACEIs: Angiotensin converting enzyme inhibitors
ACTG2: Actin gamma 2
ANOVA: One-way analysis of variance
ARBs: Angiotensin receptor blockers
a-SMA: Alpha-smooth muscle actin
BATMAN: a Bioinformatics Analysis Tool for Molecular mechANism of Traditional Chinese Medicine
BCA: Bicinchoninic acid assay
BP: Biological process
BSA: Bovine serum albumin
BUN: Blood urea nitrogen
C5orf58: Chromosome 5 open reading frame 58
CC: Cellular component
CCND1: Cyclin D1
CKD: Chronic kidney disease
DAB: Diaminobenzidine
DEGs: Differentially expressed genes
DL: DrugLikeness
EDTA: Ethylene diamine tetraacetic acid
EGFR: Epidermal growth factor receptor
ELISA: Enzyme-linked immunosorbent assay
EMT: Epithelial-to-mesenchymal transition
ERK: Extracellular regulated protein kinases
ESR1: Estrogen receptor 1
ESRD: End-stage renal disease
FH: FZHY high-dose
FL: FZHY low-dose
FOS: FBJ murine osteosarcoma viral oncogene homolog/Fos proto-oncogene
FZHY: Fuzheng Huayu formula/decoction/recipe
GAPDH: Glyceraldehyde-3-phosphate dehydrogenase
GEO: Gene expression omnibus
GO: Gene ontology
HBA1: Hemoglobin subunit alpha 1
HBA2: Hemoglobin subunit alpha 2
HBD: Hemoglobin subunit delta
H-E: Hematoxylin-eosin
IACUC: Institutional laboratory animal care and use committee
IHC: Immunohistochemistry
IL-1β: Interleukin-1 beta
IL4I1: Interleukin 4 induced 1
IL-6: Interleukin-6
INS: Insulin
ITLN1: Intelectin 1
JNK/MAPK8: Mitogen-activated protein kinase 8
KDIGO: Kidney disease improving global outcomes
KEGG: Kyoto encyclopedia of genes and genomes
MAPKs: Mitogen-activated protein kinase family
MF: Molecular function
MYC: MYC proto-oncogene
NAC: N-acetylcysteine
NCBI: National center for biotechnology information
OB: Oral bioavailability
OMIM: Online mendelian inheritance in man
OR5L2: Olfactory receptor family 5 subfamily L member 2
PBS: Phosphate buffer saline
PBST: Phosphate buffer saline with Tween® 20
P-ERK: Phospho-extracellular regulated protein kinases
P-JNK: Phospho-mitogen-activated protein kinase 8
PMSF: Phenylmethylsulfonyl fluoride
PPI: Protein-protein interaction
qRT-PCR: Quantitative real-time PCR
RIPA: Radio immunoprecipitation assay
RPS4Y1: Ribosomal protein S4 Y-linked 1
RPS4Y2: Ribosomal protein S4 Y-linked 2
Scr: Serum creatinine
SD: Standard deviation
SDS: Sodium dodecyl sulfate
SDS-PAGE: Sodium dodecyl sulfate polyacrylamide gel electrophoresis
SFDA: State food and drug administration
SPF: Specific pathogen Free
SPSS: Statistical product service solutions
STRING: Search tool for the retrieval of interaction gene/proteins
TBS: Tris buffered saline
TBST: Tris buffered saline with Tween® 20
TCM: Traditional Chinese Medicine
TCMSP: Traditional Chinese Medicine systems pharmacology database and analysis platform
TMB: 3, 3′,5 ,5′-Tetramethylbenzidine
TNF-α: Tumor necrosis factor-alpha
UUO: Unilateral ureteral obstruction
VEGFA: Vascular endothelial growth factor A
WB: Western blot
