# Supplementary material for: Pharmacological mechanisms of Fuzheng Huayu formula for Aristolochic acid I–induced kidney fibrosis through network pharmacology
Source: Front Pharmacol. 2022 Dec 8;13:1056865. doi: 10.3389/fphar.2022.1056865 (PMC9779930; doi:10.3389/fphar.2022.1056865)
Supplement: Supplementary file 2 [file Table1.docx]

Table S1. Validation of the top 10 differentially expressed genes

|  | INS | IL6 | VEGFA | EGFR | CASP3 | MAPK8 | MYC | ESR1 | FOS | CCND1 |
| --- | --- | --- | --- | --- | --- | --- | --- | --- | --- | --- |
| N | 1.00±0.02 | 1.18±0.21 | 0.99±0.07 | 0.85±0.15 | 1.16±0.16 | 1.10±0.15 | 1.04±0.10 | 0.82±0.20 | 1.13±0.51 | 0.85±0.17 |
| 4w | 2.55±2.72^**^ | 1.32±0.36 | 1.75±0.60^*^ | 2.22±0.55^**^ | 3.45±1.28^**^ | 2.50±0.59^**^ | 2.24±0.47^**^ | 0.96±0.08 | 7.47±0.31^**^ | 2.62±1.74^**^ |
| 8w | 0.61±0.05 | 1.54±0.13^**^ | 0.46±0.08 | 0.60±0.11 | 1.18±0.05 | 0.89±0.14 | 2.62±0.15^**^ | 0.43±0.06^**^ | 7.84±1.28^**^ | 0.55±0.13 |
| 12w | 1.04±0.25 | 3.71±1.48^**^ | 0.58±0.07 | 0.66±0.21 | 1.57±0.15 | 1.44±0.07 | 3.54±0.44^**^ | 0.60±0.07 | 10.70±1.01^**^ | 0.64±0.16 |

Note: **P* < 0.05, compared with normal group; ***P* < 0.01, compared with normal group.
