# Supplementary figures and images for: Pharmacological mechanisms of Fuzheng Huayu formula for Aristolochic acid I–induced kidney fibrosis through network pharmacology
Source: Front Pharmacol. 2022 Dec 8;13:1056865. doi: 10.3389/fphar.2022.1056865 (PMC9779930; doi:10.3389/fphar.2022.1056865)

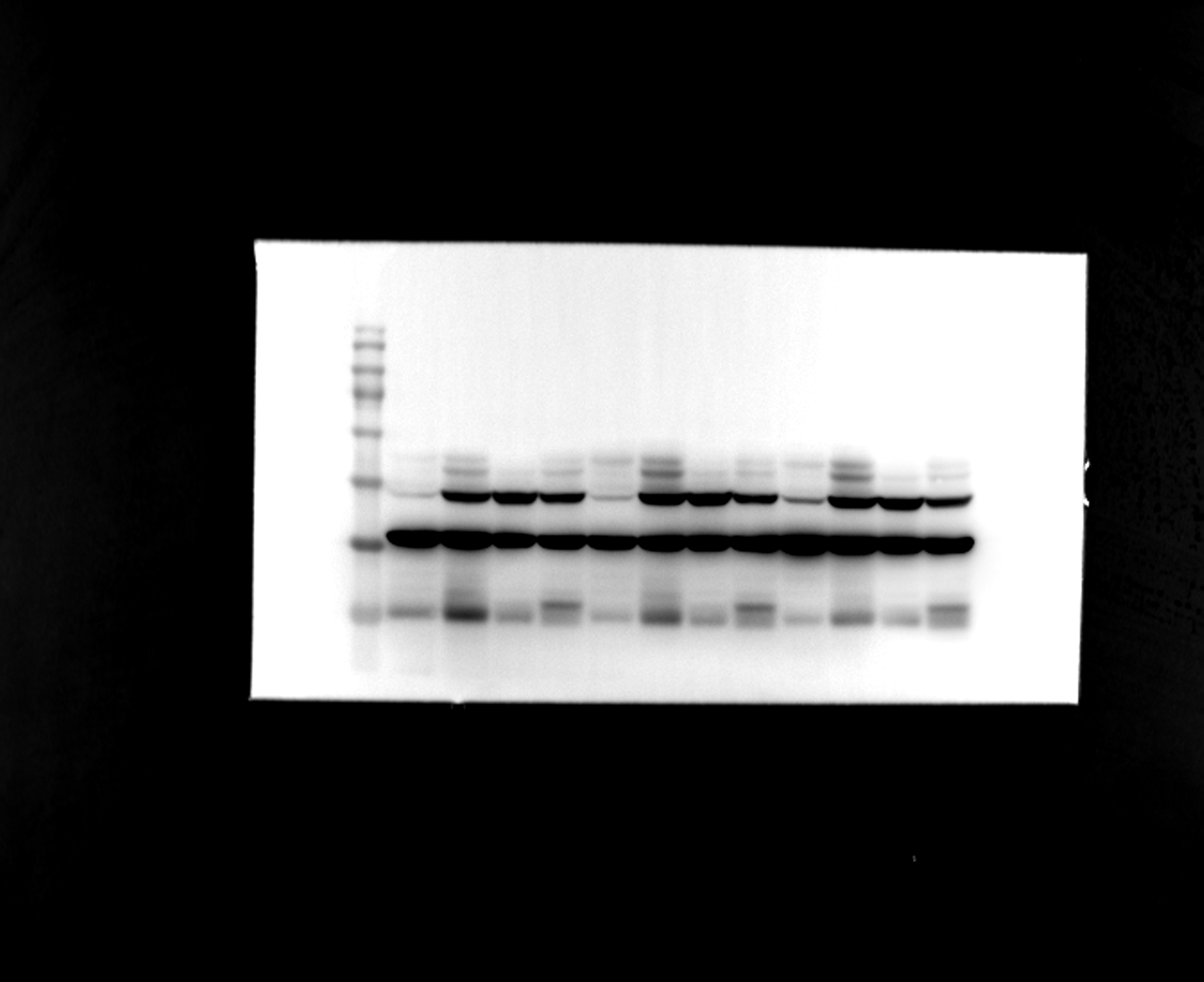

Supplement: Supplementary file 4 [file DataSheet1.zip › The original image file for the blots/Fig.4C mouse(dyna-)-AAI-ASMA+GAPDH.Tif]

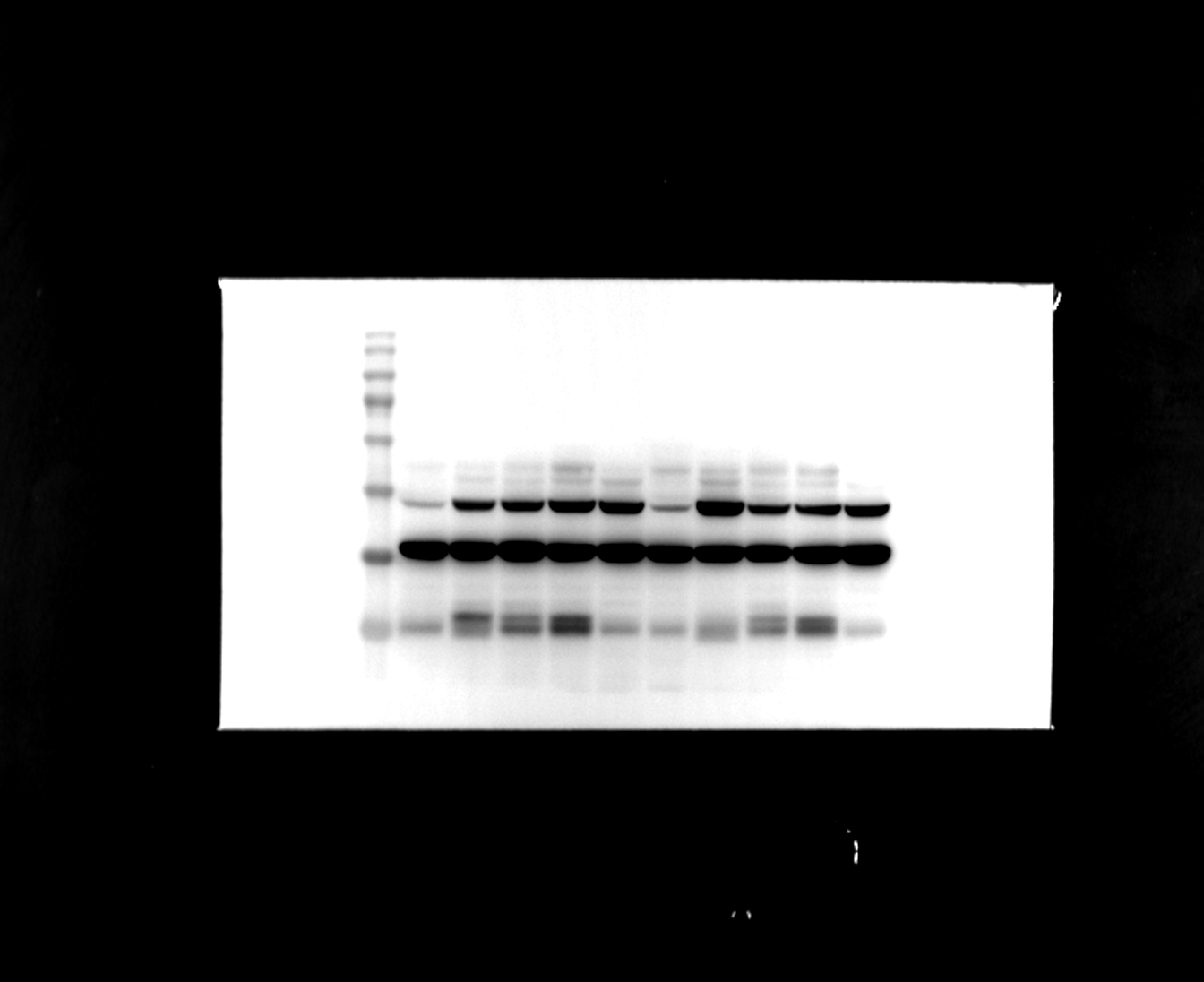

Supplement: Supplementary file 4 [file DataSheet1.zip › The original image file for the blots/Fig.4C mouse(phar-)-AAI-ASMA+GAPDH.Tif]

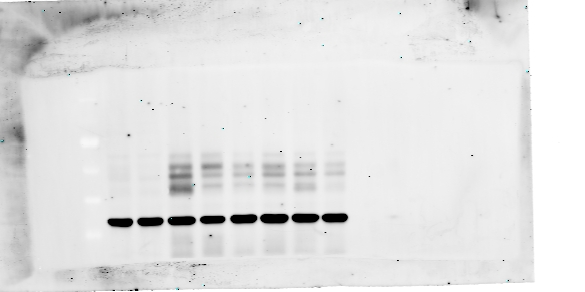

Supplement: Supplementary file 4 [file DataSheet1.zip › The original image file for the blots/Fig.5B mouse(dyna-)-AAI-FOS GAPDH.jpg]

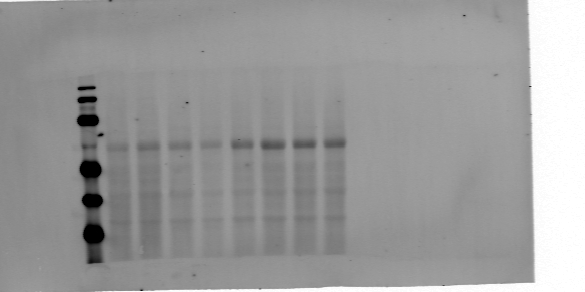

Supplement: Supplementary file 4 [file DataSheet1.zip › The original image file for the blots/Fig.5B mouse(dyna-)-AAI-FOS.jpg]

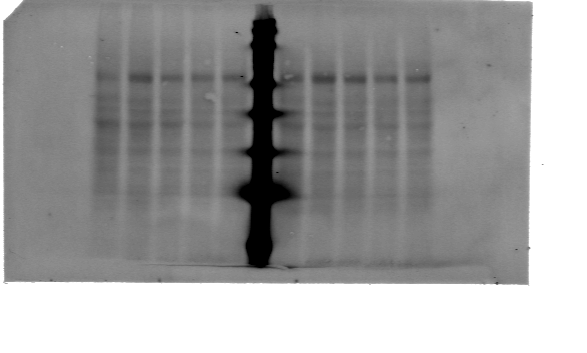

Supplement: Supplementary file 4 [file DataSheet1.zip › The original image file for the blots/Fig.5B mouse(phar-)-AAI-FOS.tif]

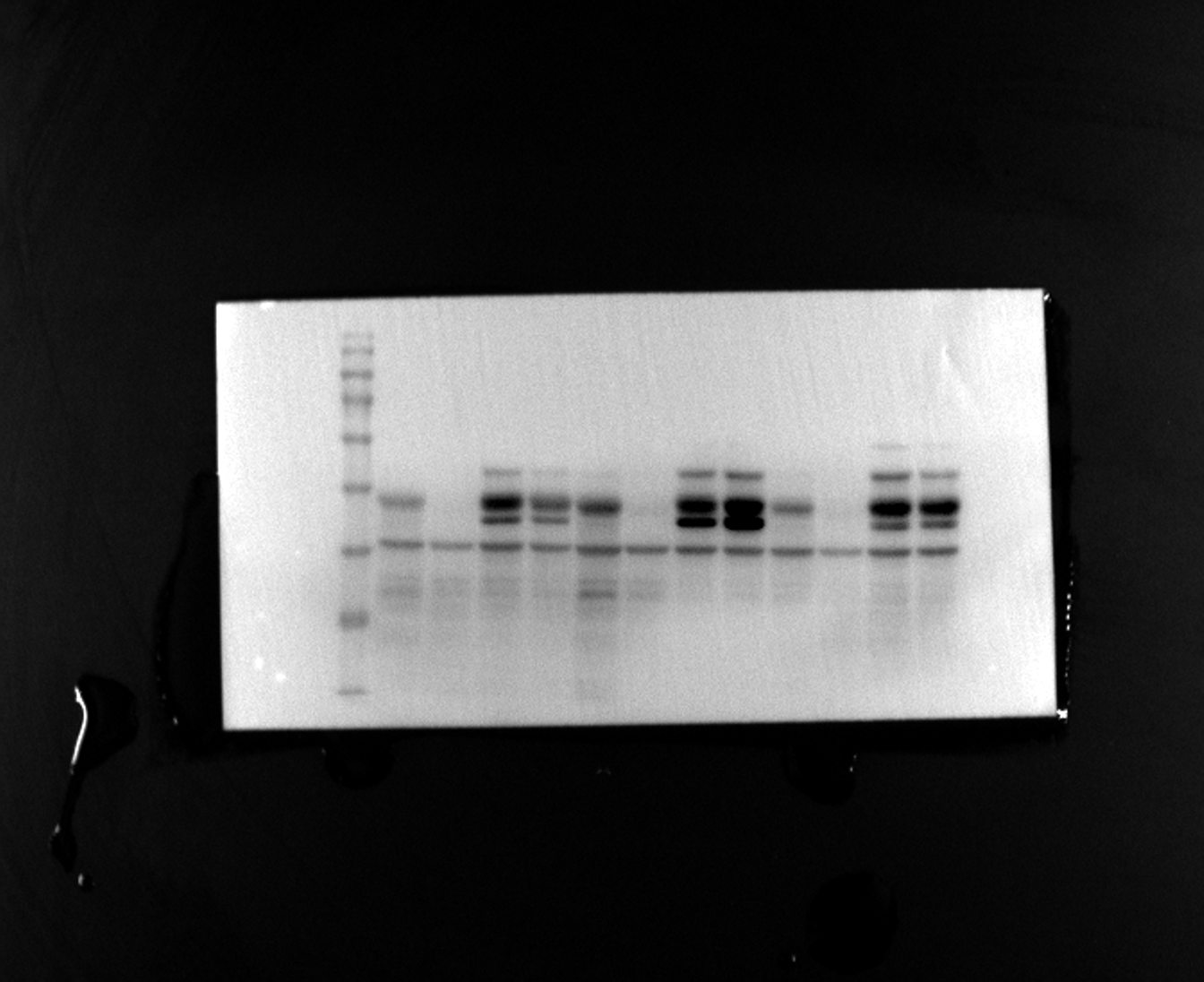

Supplement: Supplementary file 4 [file DataSheet1.zip › The original image file for the blots/Fig.6A mouse(dyna)-AAI-P-JNK+GAP.Tif]

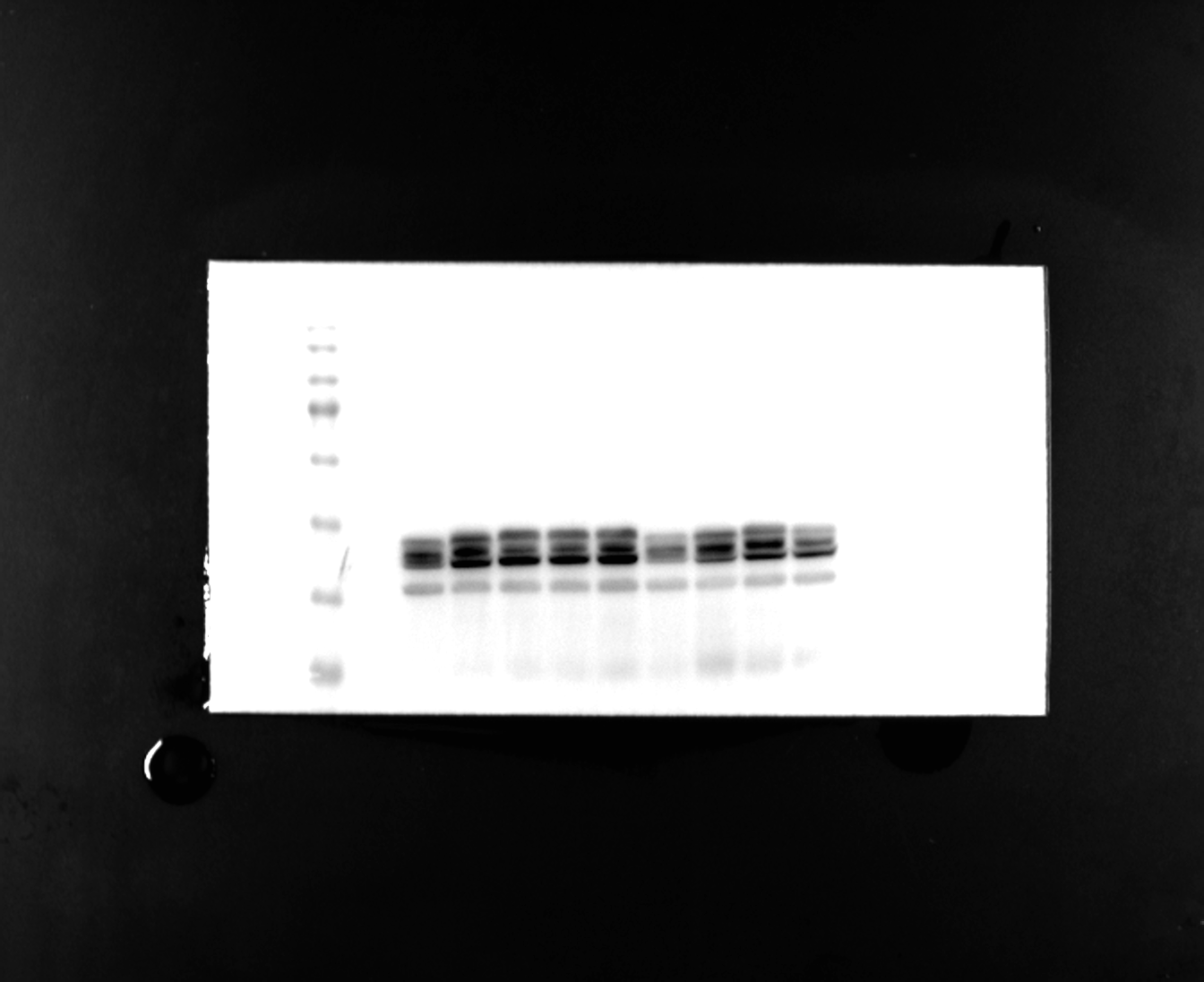

Supplement: Supplementary file 4 [file DataSheet1.zip › The original image file for the blots/Fig.6A mouse(dyna+phar)-AAI-ERK.Tif]

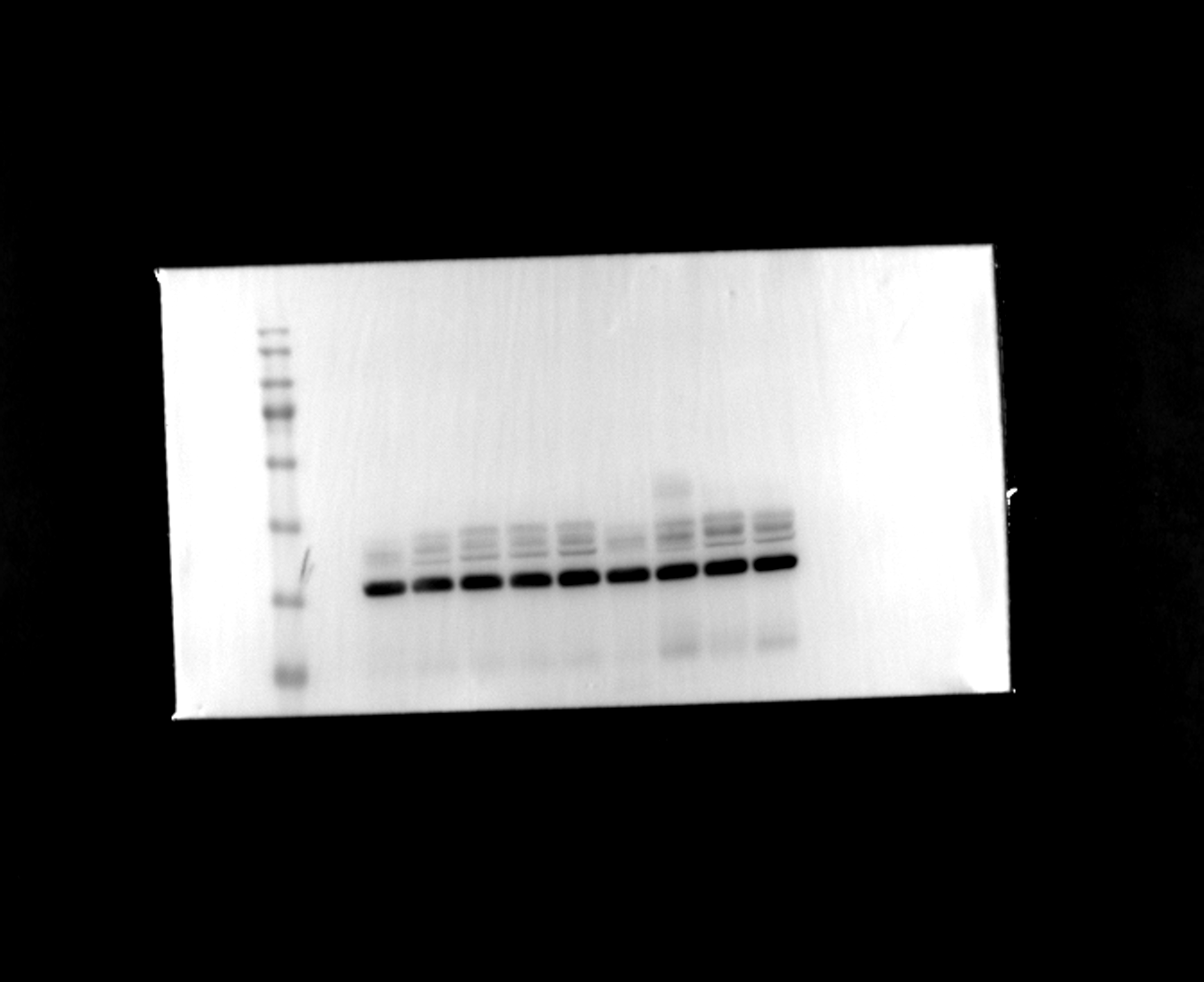

Supplement: Supplementary file 4 [file DataSheet1.zip › The original image file for the blots/Fig.6A mouse(dyna+phar)-AAI-ERK-GAPDH.Tif]

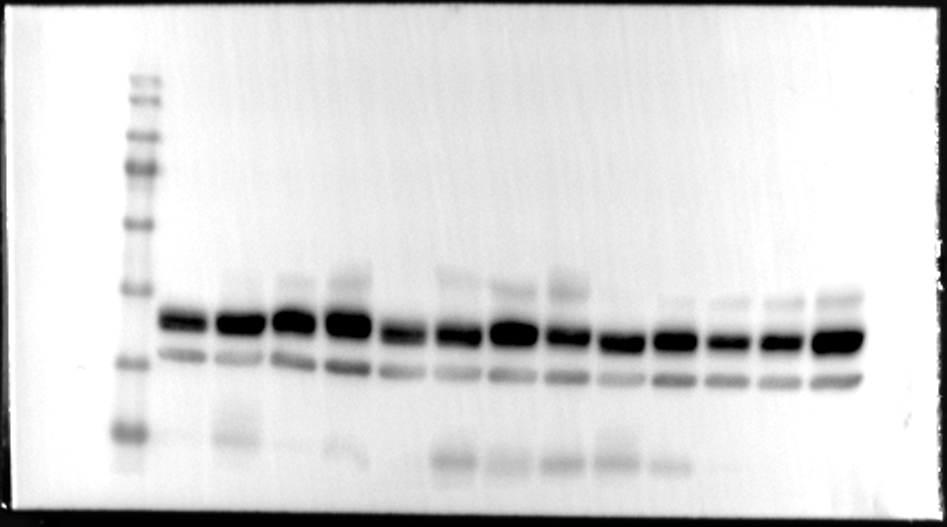

Supplement: Supplementary file 4 [file DataSheet1.zip › The original image file for the blots/Fig.6A mouse(dyna+phar)-AAI-JNK.Tif]

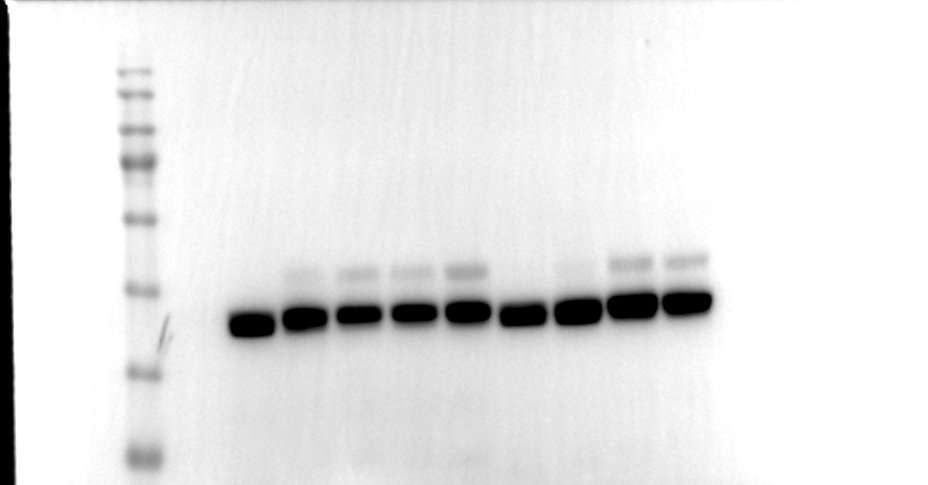

Supplement: Supplementary file 4 [file DataSheet1.zip › The original image file for the blots/Fig.6A mouse(dyna+phar)-AAI-JNK-2.Tif]

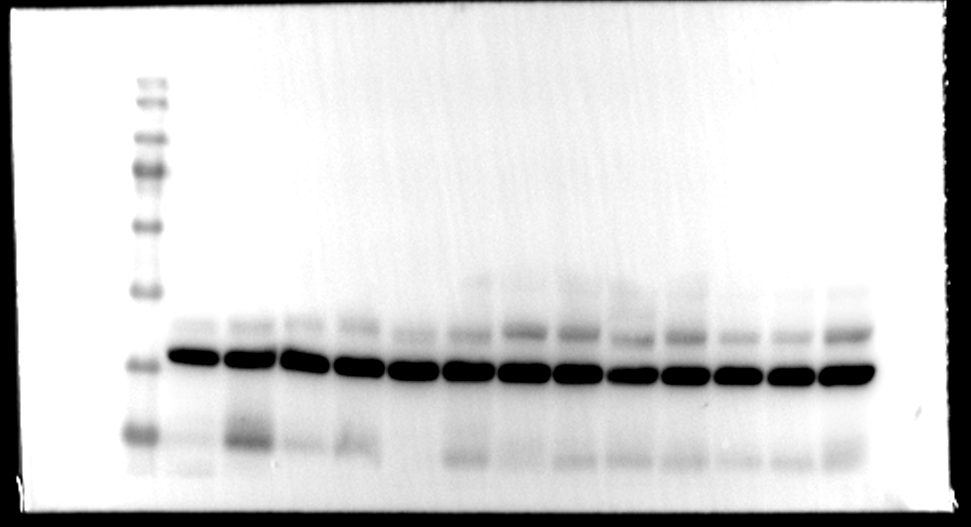

Supplement: Supplementary file 4 [file DataSheet1.zip › The original image file for the blots/Fig.6A mouse(dyna+phar)-AAI-JNK-GAPDH.Tif]

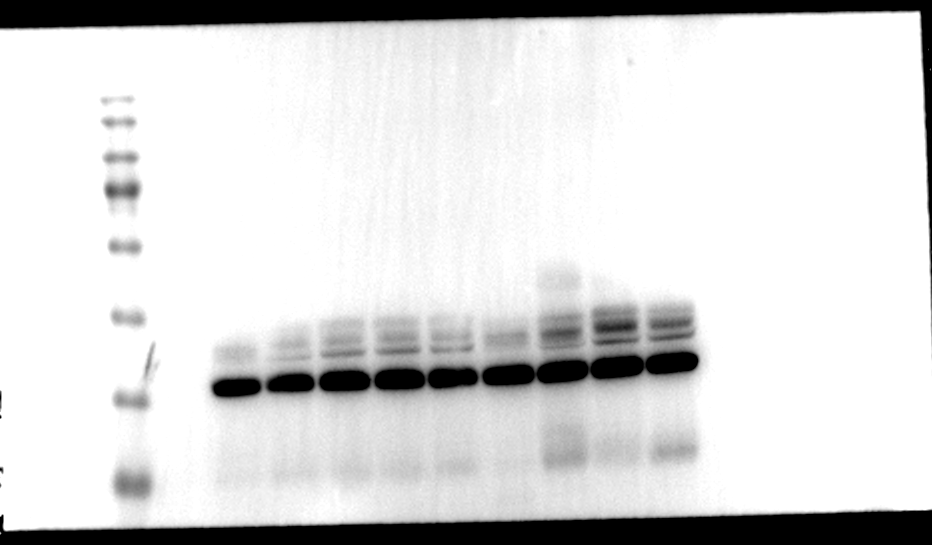

Supplement: Supplementary file 4 [file DataSheet1.zip › The original image file for the blots/Fig.6A mouse(dyna+phar)-AAI-JNK-GAPDH-2.Tif]

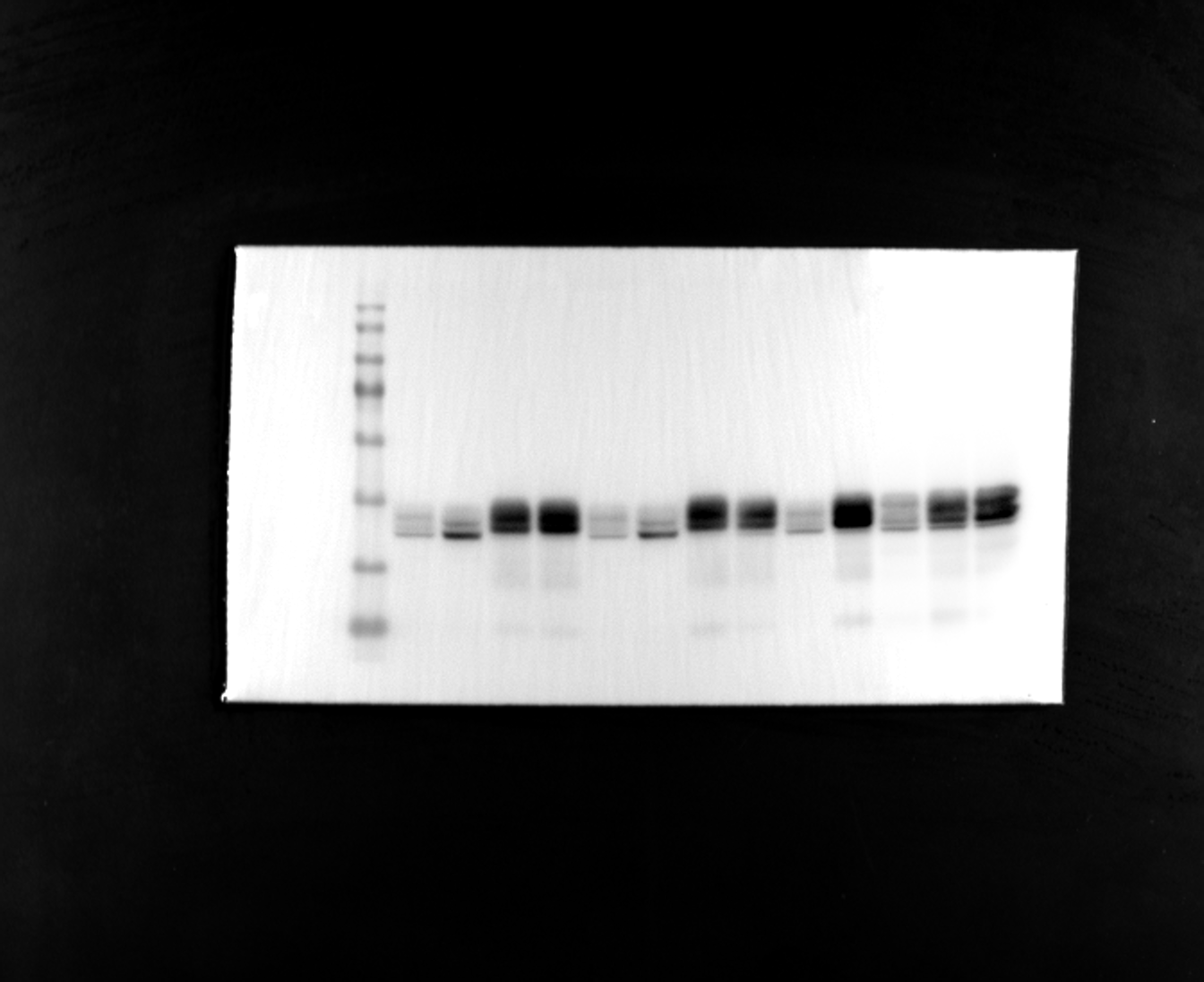

Supplement: Supplementary file 4 [file DataSheet1.zip › The original image file for the blots/Fig.6A mouse(dyna+phar)-AAI-P-ERK.Tif]

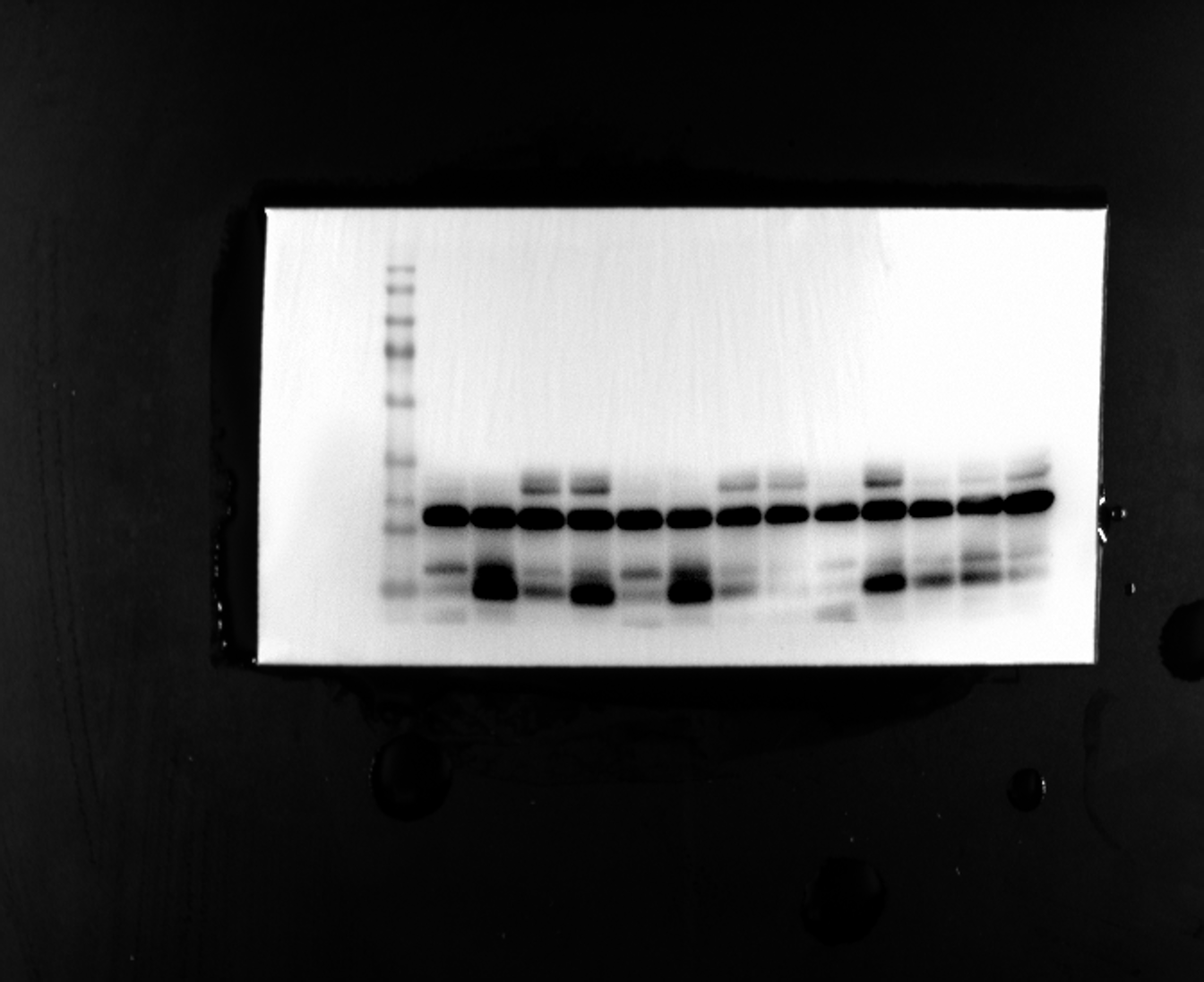

Supplement: Supplementary file 4 [file DataSheet1.zip › The original image file for the blots/Fig.6A mouse(dyna+phar)-AAI-P-ERK-GAPDH.Tif]

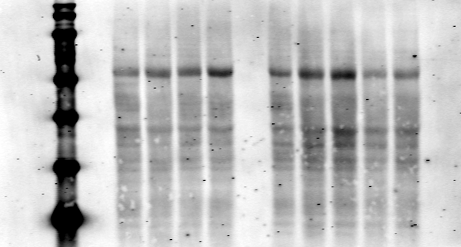

Supplement: Supplementary file 4 [file DataSheet1.zip › The original image file for the blots/Fig.6A mouse(dyna+phar)-AAI-P-FOS.tif]

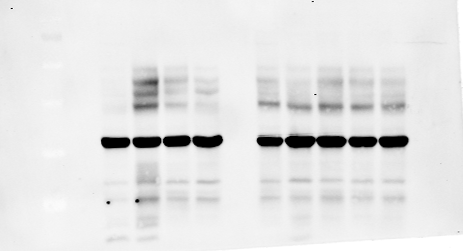

Supplement: Supplementary file 4 [file DataSheet1.zip › The original image file for the blots/Fig.6A mouse(dyna+phar)-AAI-P-FOS-GAPDH.tif]

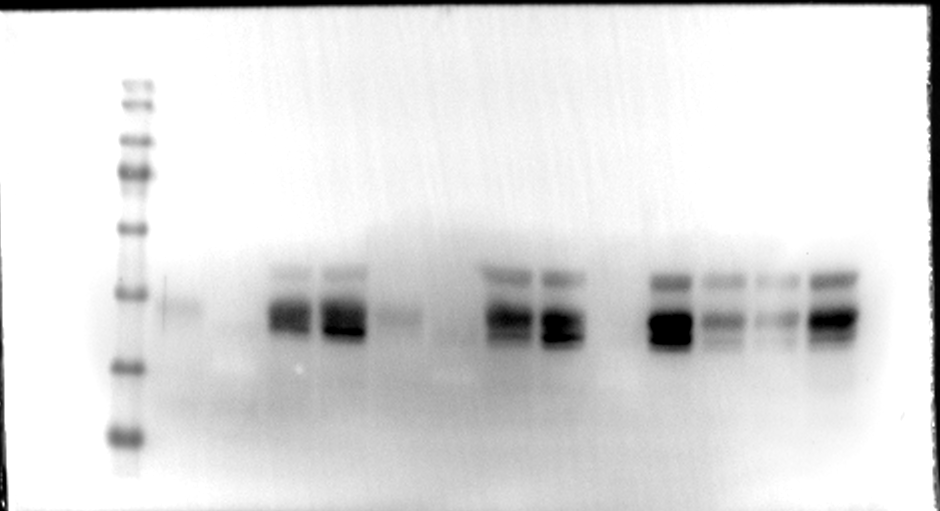

Supplement: Supplementary file 4 [file DataSheet1.zip › The original image file for the blots/Fig.6A mouse(dyna+phar)-AAI-P-JNK.Tif]

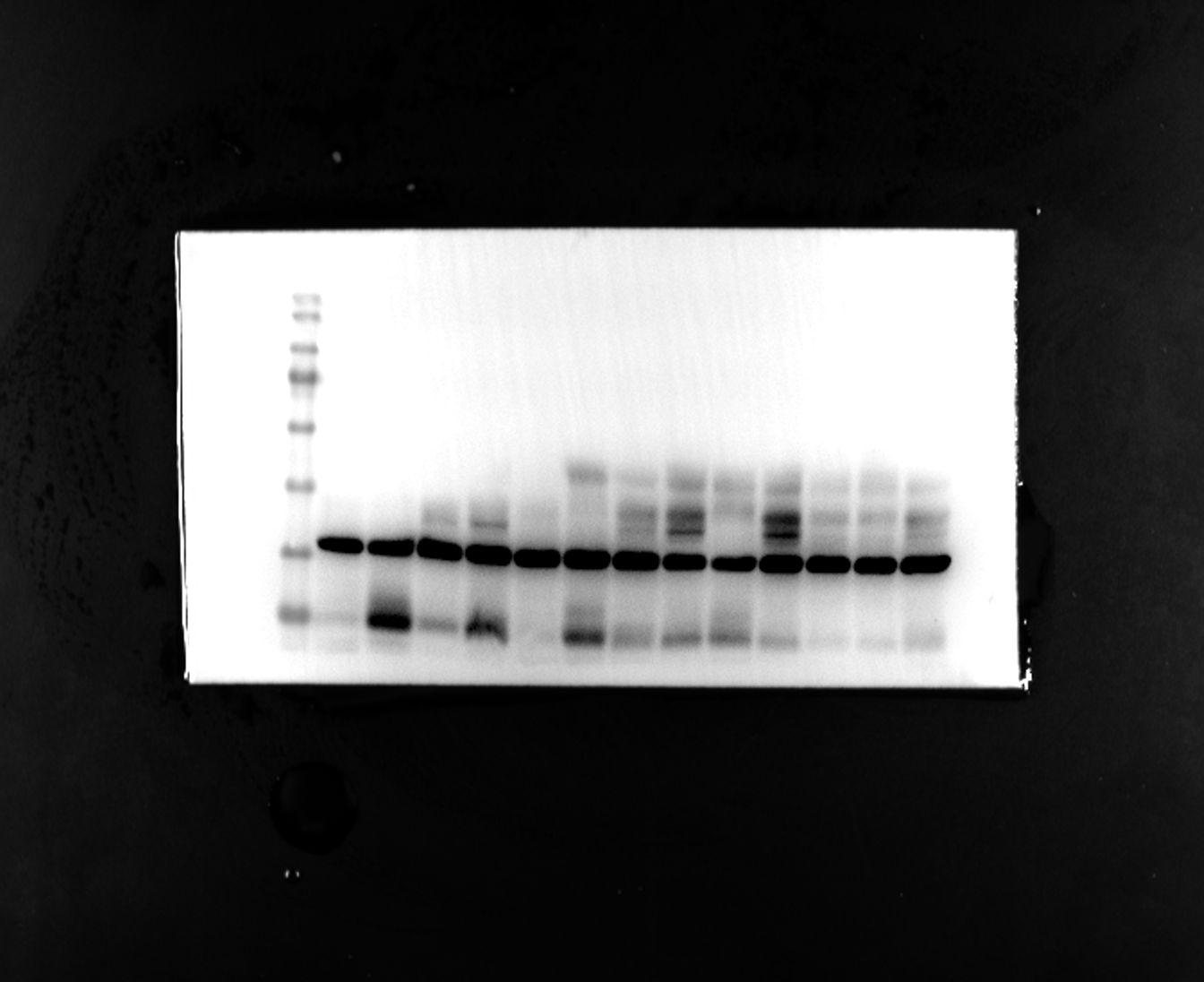

Supplement: Supplementary file 4 [file DataSheet1.zip › The original image file for the blots/Fig.6A mouse(dyna+phar)-AAI-P-JNK-GAP.Tif]

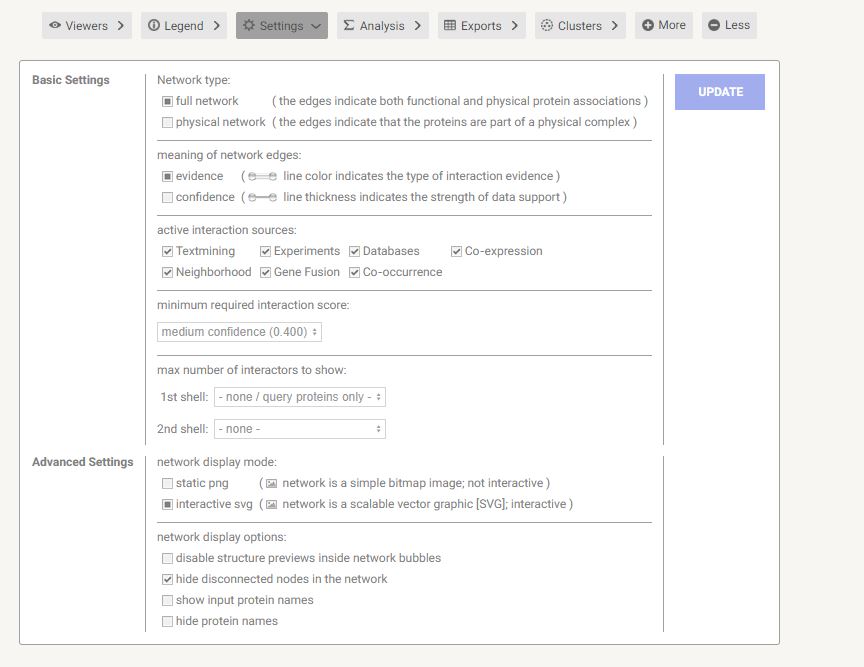

Supplement: Supplementary file 5 [file DataSheet2.zip › the original source data of Figures 1-3/PPI/PPI╖╓╬÷╔Φ╓├▓╬╩2.JPG]

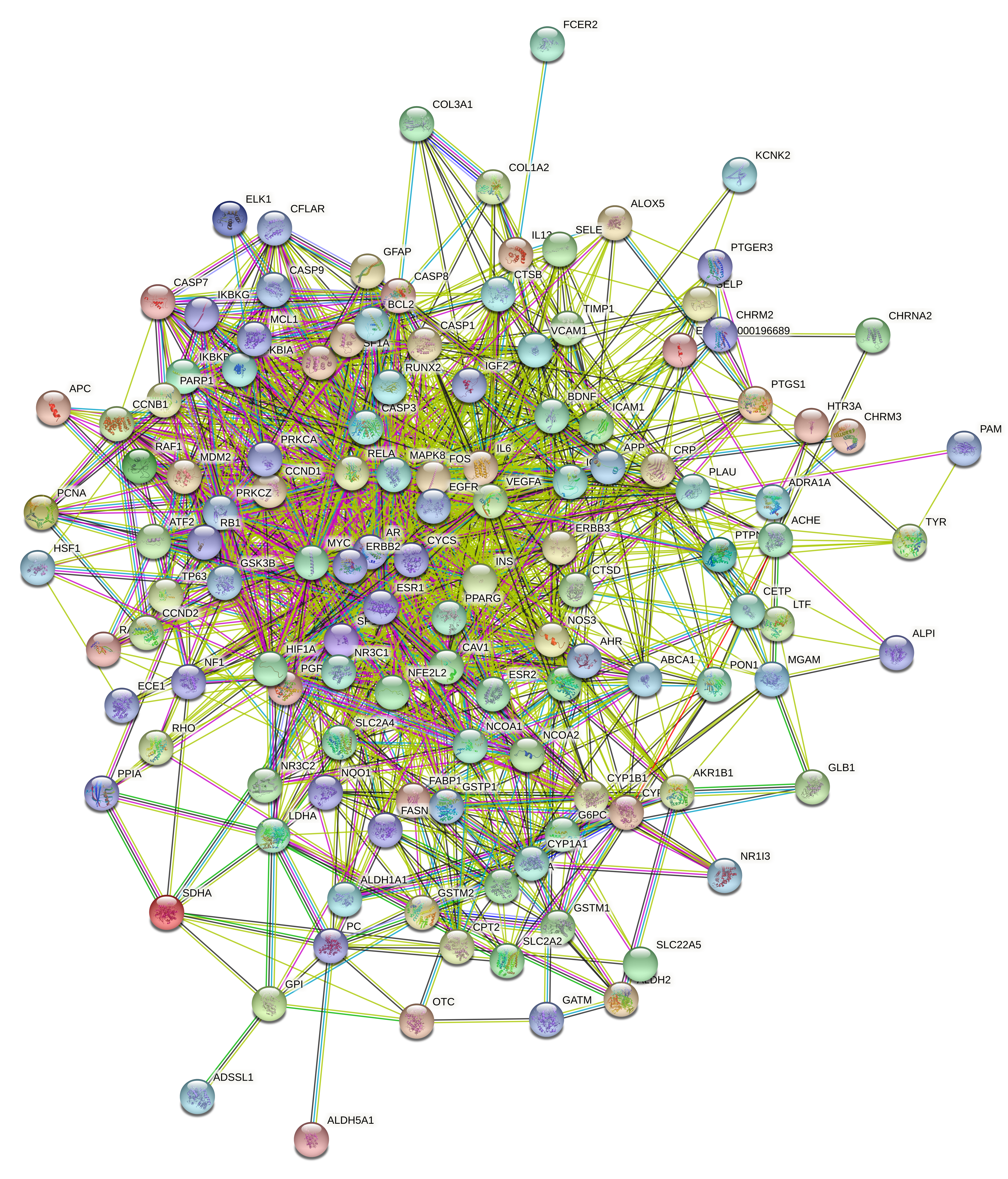

Supplement: Supplementary file 5 [file DataSheet2.zip › the original source data of Figures 1-3/PPI/string_hires_image.png]
